# Multivariate Time Series Information Bottleneck

**DOI:** 10.3390/e25050831

**Published:** 2023-05-22

**Authors:** Denis Ullmann, Olga Taran, Slava Voloshynovskiy

**Affiliations:** Faculty of Science, University of Geneva, CUI, 1227 Carouge, Switzerland; denis.ullmann@unige.ch (D.U.);

**Keywords:** multiple time series, forecasting method, information bottleneck, entropy, KL-divergence, mutual information, deep models, RNN, U-Net, partial convolutions

## Abstract

Time series (TS) and multiple time series (MTS) predictions have historically paved the way for distinct families of deep learning models. The temporal dimension, distinguished by its evolutionary sequential aspect, is usually modeled by decomposition into the trio of “trend, seasonality, noise”, by attempts to copy the functioning of human synapses, and more recently, by transformer models with self-attention on the temporal dimension. These models may find applications in finance and e-commerce, where any increase in performance of less than 1% has large monetary repercussions, they also have potential applications in natural language processing (NLP), medicine, and physics. To the best of our knowledge, the information bottleneck (IB) framework has not received significant attention in the context of TS or MTS analyses. One can demonstrate that a compression of the temporal dimension is key in the context of MTS. We propose a new approach with partial convolution, where a time sequence is encoded into a two-dimensional representation resembling images. Accordingly, we use the recent advances made in image extension to predict an unseen part of an image from a given one. We show that our model compares well with traditional TS models, has information–theoretical foundations, and can be easily extended to more dimensions than only time and space. An evaluation of our multiple time series–information bottleneck (MTS-IB) model proves its efficiency in electricity production, road traffic, and astronomical data representing solar activity, as recorded by NASA’s interface region imaging spectrograph (IRIS) satellite.

## 1. Introduction

The scope of this work lies at the intersection of several domains, offering contributions to information theory (IT) applied to machine learning (ML), applied astrophysics, and computer vision (CV). Recently, non-recurrent models, such as transformers [1], among others [2,3], have been used to accurately predict forecasts for multivariate time series (MTS). The training of these models is guided by a proposed information–theoretical approach. This study supports the validity of some of these models with an IT approach describing the IB in the context of time series (TS). A CV-based model using partial convolution [4] with an MTS forecasting goal is presented and the link with the IB principle is proved. The approach was tested on astrophysical production, electricity production, and road traffic data. MTS, CV, and IT metrics show the empirical effectiveness of the proposed idea.

TS and MTS predictions are among the key applications of ML. They enable models to forecast the future evolution of data over time, where the time flow is represented as a single scalar for TS and a multi-dimensional vector for MTS. Meteorology, finance, online purchases, epidemic spread, and space weather forecasting are all examples of areas with great interest. Even a marginal improvement, as small as a tenth of a percent, can have a significant monetary or scientific impact. TS and MTS predictions are domains where models, such as recurrent neural networks (RNNs) [5] and long-short-term-memory (LSTM) [6], were historically developed to comply with the specificities of the temporal dimension. Although these models have similarities with classical CV models, they are part of a separate group of models that aim to mimic human memory and attention mechanisms.

Historically, one-step-forward models were formalized before multi-step-forward models. For the former, the model predicts one step of time after a known time series. Before the rise of deep models, time series decompositions, regressions [7,8,9,10], moving averages [11], exponential smoothing techniques [12], and ARIMA [13] models were designed to forecast the most probable outcome of a time step following the given time steps in a series. Later, deep models advocated for learning a larger number of regression parameters through gradient descent [14]. RNNs face a vanishing gradient problem when they consist of more than three layers. LSTMs, a family of RNNs, aim to mimic the memory and synaptic functioning of human brains, as well as solve the vanishing gradient issue, at the cost of a possible explosion of the gradients. Finally, the more recent gated recurrent unit (GRU) [15] is an intermediate version of RNNs that works efficiently in more cases compared to classical RNNs and LSTMs.

The most recent challenges of forecasting with deep models include (a) achieving high-efficiency prediction in the context of MTS, where each time step consists of a multi-dimensional vector, with multi-step-ahead forecasts, where the model predicts multiple time steps ahead in one run, (b) interpretability, and (c) predicting the errors at each forecasted time step. The error prediction is often performed by integrating them as a joint time series that the model has to predict in parallel to the targeted time series [2]. Another option relies on stochastic predictions, where the possible forecast at each time is modeled by probabilistic distributions whose parameters are predicted by the model [2]. Interpretability is often obtained by explicit time representations or decompositions [16,17,18], as well as hierarchically built models that are supposed to learn classical time series decompositions by trend and seasonality [3]. To our knowledge, the most significant recent performance gains have been obtained by models that first construct a joint embedding representation of the time and space dimensions, along with compression and decompression techniques [2,3,17,18,19,20], which are afterward fed into a version of RNN, graph neural networks (GNNs) [21] or self-attention network-like transformers [17], at very high memory costs [18,20].

On the other hand, from the CV family of deep models, the objectives of *image inpainting* [4,22,23,24] is very similar to TS forecasting. Both attempt to recover some missing data from the information provided by two correlated known dimensions: pixel coordinates for CV and temporal–spatial for MTS. For TS and MTS, the observed temporal evolution of some data provides information to the model to forecast how these data will evolve in the future [25,26]. For image inpainting, the given parts of the image serve as prior information that aids the model in reconstructing the missing parts [24].

Recent works on images denoising [27] and inpainting [4] have shown a high capability to capture prior distributions of images and restore masked or noisy images with high accuracy. They measure accuracy in various ways, including classical Manhattan or Frobenius norms, more advanced styles [28], perceptual losses [29], and human assessments such as the mean opinion score (MOS) [30]. All of these methods use the U-Net architecture [31], with partial convolutions [4], which are particularly efficient for learning outputs that are close to the inputs in terms of similar pixels.

U-Net is a deep convolutional network that was originally designed for image segmentation [31]. It can be sketched by the following Markov chain: 
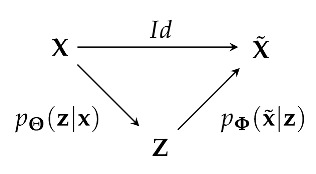
 where Z is a latent representation, $p_{\Theta }\left(z|x\right)$ is an encoding part modeled by reducing the time dimension through successive strided convolution layers, and $p_{\Phi }\left(\tilde{x}|x\right)$ is a decoding part performed by an architecture symmetric to $p_{\Theta }\left(z|x\right)$, such that the posterior $\tilde{X}$ has the same shape as the prior X. U-Net also allows a direct flow of information with an identity mapper Id between X and $\tilde{X}$, also referred to as the skipping layer. This direct flow of information between the prior and the posterior allows for easy reconstruction of the prior image while the information flow through the latent Z is responsible for the image segmentation objective. Skipping layers are also present between symmetrical hidden layers of the encoder and the decoder. Without the skipping layers, the U-Net is reduced to an autoencoder (AE) structure [32] sketched by the Markov chain $X\to Z\to \tilde{X}$ acting as a principal component analysis dimensional reduction of X in Z.

Considering the spectral time sequences as images, one can demonstrate that image processing based on generative machine learning techniques can capture the temporal patterns of the physics or logic behind the spatial data, enabling the prediction of short-term evolution. Therefore, our objective is to predict time sequences efficiently with the support of the IB principle rather than classical time sequence modelings, such as LSTM or RNN. The problem with time sequence prediction is, in this way, very similar to *image inpainting* or an *extension* problem [4,22,23].

Recently, IT approaches have formalized deep models through IB [33]. This shows that deep models are guided to find the most informative yet compressed representations for given tasks. Deep models must compress the input information into a format that ideally contains only sufficient statistics to recover posterior targeted data. To our knowledge, very few past works [34,35,36,37] have attempted to describe the IB principle in the TS and MTS contexts, whereas an extensive body of literature exists that focuses on deriving the IB principle in CV models [33,38,39,40,41,42].

Very few past works have used IT to design or explain the TS and MTS forecasting models that they proposed. Some attempted to estimate the entropy of TS or MTS in order to quantify their variability [34,35,37]. More interestingly, the IB principle was formulated in the RNN context but without compression of the time dimension, such that only the information present at each time step was compressed and decompressed [36]. Their work claims that each time step can be formulated by its own IB principle and that a time series with N time steps can be modeled by N IB steps. In our work, we claim that the compression of time is key for efficient forecasting and most of the existing models are realizations of a single IB principle with the compression of time and spatial dimensions.

From an information–theoretical point of view, Tishby [33] proposed the information bottleneck principle (IB), which aims to compress the input X and filter out all task-irrelevant information while preserving sufficient statistics in the bottleneck Z; this was in order to decode the compressed representation into the task-specific representation, denoted as $\tilde{X}$ in this context. The goal of the model is to find the parameters of the compression encoder Θ and the decoding Φ by solving the following optimization problem:

(1) $$\left(\hat{\Theta },\hat{\Phi }\right)=\underset{\begin{array}{c} \left(\Theta ,\Phi \right)\\ I_{\Phi }\left(Z;\tilde{X}\right)\geq \alpha \end{array}}{\mathrm{argmin}}I_{\Theta }\left(X;Z\right),$$

where $I_{\Theta }\left(X;Z\right)$ represents Shannon’s mutual information between X and Z, which is parameterized by the parameters Θ of the network $f_{\Theta }\left(\cdot \right)$ mapping X to Z, defined by:

(2) $$I_{\Theta }\left(X;Z\right)=E_{p(X,Z)}\left[\log_{2}\frac{p(X,Z)}{p(X),p(Z)}\right],\;\mathrm{where}\;z∼p_{\Theta }\left(z|x\right)\;\mathrm{for}\;\mathrm{all}\;x∼X.$$

α represents the lower bound on the mutual information between the genuine $\tilde{X}$ and the compressed Z. This lower bound ensures sufficient statistics of the genuine in the compressed Z in order to allow the decoder to decode $\tilde{X}$. Equation (1) can also be refined with a Lagrange multiplier β, such that the parameters of the compression and decompression are solutions of:

(3) $$\left(\hat{\Theta },\hat{\Phi }\right)=\underset{\left(\Theta ,\Phi \right)}{\mathrm{argmin}}\underset{L(\Theta ,\Phi )}{\underset{︸}{I_{\Theta }\left(X;Z\right)-\beta I_{\Phi }\left(Z;\tilde{X}\right)}}.$$

Past works [43,44,45,46] have studied the IB of AEs, often by empirically estimating the information plane (IP), i.e., the temporal graph of the training relation between mutual information, I(X;Z) and $I(Z;\tilde{X})$. For all studies, the estimation of mutual information is not exact and requires the tuning of some hyperparameters. In [43,46], the authors studied the IP at training times for different types of AEs. They show that sparse autoencoders (SAEs) significantly compress the information of MNIST data in the bottleneck, unlike the other AE, such as the variational autoencoder (VAE), for which the compression is not clear for the data (even though the VAE provides high constraints on the distribution of the bottleneck). More details about the variational decomposition of the IB and its variational approximations are provided in [47]. In [44], the authors studied the IP of vanilla AE on MNIST with different hyperparameters for mutual information estimation. Their work shows the compression of information at each hidden layer, extending from the input to the bottleneck layer. It also provides an interpretation of the link between the dimensions of the bottleneck and the compression of information. It shows that when the bottleneck dimensions are relatively small, compared to the entropy of the source, further compression is forced due to the limitation imposed by the bottleneck dimension. When the bottleneck dimensions are relatively large, there are no such limitations. Our broad interpretation of this outcome is that the AE training follows Shannon’s separation theorem from the joint source and channel coding theory [48] because of the large capacity of the channel formed by the AE. In [45], the authors studied the rate-distortion performance of an AE where the IB was used as the dimensionality reduction with a fixed number of noisy information channels; they applied this AE strategy to efficiently store analog data on an array of phase-change memory (PCM) devices. The IB of AEs showed efficient rate-distortion results in this context; the authors provided theoretical insights by utilizing Shannon’s separation theorem from the joint source and channel coding theory [48].

We propose a general formulation of the IB principle for MTS forecasting. We show that the U-Net architecture with source masking and an approximation of the IB loss can be regarded as a particular instance of the formulated IB principle for MTS. We provide an extensive evaluation of the proposed model on some astrophysical data of interest, and we compare the model to concurrent ones with MTS, CV, and astrophysical metrics. Interestingly, without fine-tuning, and with an approximation of the IB loss, our models based on the IB principle formulation can achieve top results on different datasets involving astrophysics solar activity prediction, electricity production, and road traffic.

One important direction of this work is the application of the IB principle to astrophysical data. The accurate prediction of solar activity, solar flares, in particular, is still an open issue. Solar flares occur as a result of the reconfiguration of magnetic fields in the corona. These energetic events accelerate highly energetic particles into space and toward the solar surface, where they cause heat and emissions in a broad range of wavelengths. Solar flares are major protagonists in space weather and can cause adverse effects, such as disruptions in communications, power grid failures on Earth, and damage to satellites and other critical infrastructures. Many attempts to predict flares exist [49,50,51,52,53], as well as works on flare detection [54] and analyses [55,56,57,58].

## 2. Methods

Forecasting models take TS data as input, noted as $X_{1:T}=\left[X_{1}:X_{T}\right]$, or MTS data, noted $X_{1:T}=\left[X_{1}:X_{T}\right]$, both of length *T*. For TS, each time step $X_{t},\;\left(t\in \left[1,\ldots ,T\right]\right)$ is scalar, whereas for MTS, each time step $X_{t}=\left[X_{t}^{1},\ldots ,X_{t}^{M}\right],\;\left(t\in \left[1,\ldots ,T\right]\right)$ is a vector of length *M*. As a consequence, $X_{1:T}$ represents a two-dimensional tensor, with the first dimension being *temporal* of length *T*, and the second typically referred to as the *spatial* dimension of length *M*.

The goal of the forecasting models is to predict the time continuation of the input data by forecasting one step ahead or multiple steps ahead; this is denoted as $X_{T+1:T+F}=\left[X_{T+1},\ldots ,X_{T+F}\right]$ for TS, and $X_{T+1:T+F}=\left[X_{T+1},\ldots ,X_{T+F}\right]$ for MTS, where *F* refers to the number of steps ahead to forecast.

In this paper, the input series $X_{1:T}$ or $X_{1:T}$ is referred to as the *prior*, the true forecast $X_{T+1:T+F}$ (or $X_{T+1:T+F}$) is referred to as *genuine*, and the forecast predicted by the model ${\tilde{X}}_{T+1:T+F}$ or ${\tilde{X}}_{T+1:T+F}$ is referred to as *posterior*. *D* refers to the training dataset, which is also denoted as ${\left\{X_{1:T+F}\right\}}_{D}$ or ${\left\{\left(X_{1:T+F},K\right)\right\}}_{D}$ when the data are labeled; *K* denotes the label, or it can be represented as K in the case of categorical vectors. Table A1 recalls most of the notations used in the paper.

### 2.1. IB-Based Optimal Compression for Time Series Forecasts

The general IB principle proposes to compress the input into a latent representation while ensuring the preservation of sufficient statistics, which are crucial for the downstream task. In the context of TS forecasting, this implies that the effective compression of the time dimension needs to be employed in order to achieve accurate forecasting. Using our notations, and according to the IB formulation of Equation (3), the goal of a model is to find the parameters of the compression encoder Θ and of decoding Φ by solving the following optimization problem:

(4) $$\left(\hat{\Theta },\hat{\Phi }\right)=\underset{\left(\Theta ,\Phi \right)}{\mathrm{argmin}}\underset{L(\Theta ,\Phi )}{\underset{︸}{I_{\Theta }\left(X_{1:T};Z_{ib\_tr}\right)-\beta I_{\Phi }\left(Z_{ib\_tr};X_{T+1:T+F}\right)}},$$

where the input $X_{1:T}$ represents the previous *T* time steps of values, and the output $X_{T+1:T+F}$ represents the subsequent *F* time steps. As a consequence, the bottleneck variable $Z_{ib\_tr}$ should then hold the necessary part of information of prior time steps 1:*T* for the model to be able to forecast the time steps T+1:T+F. The index ib_tr explicitly reflects the nature of the MTS bottleneck task as an IB learning statistics of transitions 1:T→T+1:T+F. Taking inspiration from works [39], Proof of Equation (5) in the Appendix A shows that an upper bound $\tilde{L}\left(\Theta ,\Phi \right)$ on the loss L(Θ,Φ) of Equation (3) can be reduced as follows:

(5) $$\tilde{L}\left(\Theta ,\Phi \right)=H_{p_{\Theta }}\left(Z_{ib\_tr}\right)-H_{p_{\Theta }}\left(Z_{ib\_tr}|X_{1:T}\right)+\beta H_{p_{\Theta ,\Phi }}\left(X_{T+1:T+F}|Z_{ib\_tr}\right),$$

where $p_{\Theta }\left(Z_{ib\_tr}\right)=E_{p_{D}}\left[p_{\Theta }\left(Z_{ib\_tr}|X_{1:T}\right)\right]$, where *D* represents the training data consisting of pairs of priors and their corresponding known forecasts: $D=\left\{\left(x_{1:T},x_{T+1:T+F}\right)∼X_{1:T+F}\right\}$ and H(.) stands for Shannon’s entropy, such that the upper bound on L(Θ,Φ) is composed by these three components:

(6) $$\begin{array}{cc} L_{1}\left(\Theta \right) & =H_{p_{\Theta }}\left(Z_{ib\_tr}\right)\\ \; & =-E_{p_{\Theta }\left(Z_{ib\_tr}\right)}\left[\log_{2}p_{\Theta }\left(Z_{ib\_tr}\right)\right],\\ L_{2}\left(\Theta \right) & =-H_{p_{\Theta }}\left(Z_{ib\_tr}|X_{1:T}\right)\\ \; & =E_{p_{\Theta }\left(X_{1:T},Z_{ib\_tr}\right)}\left[\log_{2}p_{\Theta }\left(Z_{ib\_tr}|X_{1:T}\right)\right],\\ L_{3}\left(\Theta ,\Phi \right) & =H_{p_{\Theta ,\Phi }}\left(X_{T+1:T+F}|Z_{ib\_tr}\right)\\ \; & =-E_{p_{D}\left(X_{1:T+F}\right)}\left[E_{p_{\Theta }\left(Z_{ib\_tr}|X_{1:T}\right)}\left[\log_{2}p_{\Phi }\left(X_{T+1:T+F}|Z_{ib\_tr}\right)\right]\right]. \end{array}$$

$L_{3}\left(\Theta ,\Phi \right)$ is the average cross-entropy $H(p_{\Theta }\left(Z_{ib\_tr}|X_{1:T}\right),p_{\Phi }\left(X_{T+1:T+F}|Z_{ib\_tr}\right))$. Moreover, if the decoding distribution $p_{\Phi }\left(X_{T+1:T+F}|Z_{ib\_tr}\right)$ is assumed to follow the Laplacian distribution, ref. [39] shows that the loss $L_{3}\left(\Theta ,\Phi \right)$ can be reduced into the average Manhattan distance, which is also referred to as the mean average error loss (MAE) between the genuine and the model estimation:

(7) $$\begin{array}{cc} L_{3}^{Lap}\left(\Theta ,\Phi \right) & =E_{p_{D}\left(X_{1:T+F}\right)}\left[E_{p_{\Theta }\left(Z_{ib\_tr}|X_{1:T}\right)}\left[{∥X_{T+1:T+F}-g_{\Phi }\left(Z_{ib\_tr}\right)∥}_{1}\right]\right]. \end{array}$$

**Figure 1:**
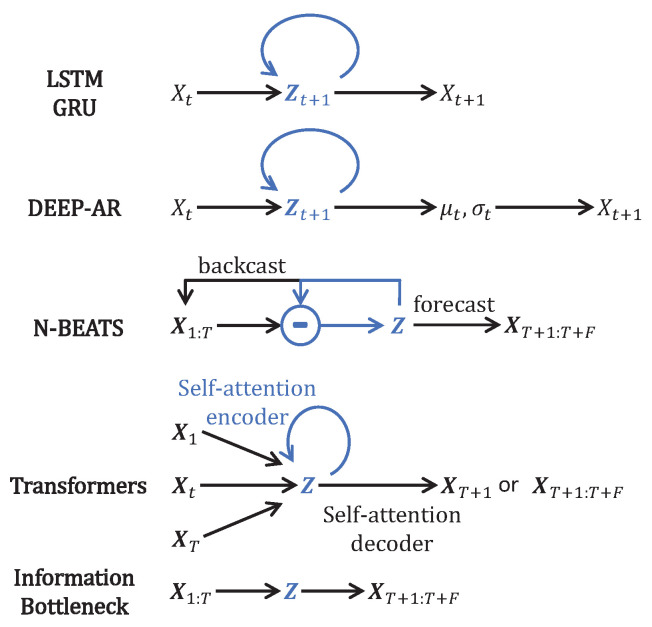
Comparison of Markov chains for a selection of deep TS predictors: the blue parts correspond to the compressed representations of the time dimension. Some of these may accept additional inputs (correlated context) but we did not include them in these diagrams because that would overload the global understanding, and the time dimension is compressed in the same way. A bold X is used when the model accepts vectors as input.

Throughout the years, different forecasting models have been proposed; the MAE of $L_{3}^{Lap}\left(\Theta ,\Phi \right)$ has been used for training the initial models and for evaluating the performances of the forecasting models. We show in Figure 1 how existing models design a bottleneck Z that compresses the time dimension, similar to $Z_{ib\_tr}$ in Equation (4). The following paragraphs briefly explain the time compression and a few differences between the models selected in Figure 1.

LSTM [6], GRU [15], and DEEP-AR [2] operate with an RNN [5] over the time dimension, which is compressed in the hidden memory channel Z. The constituting cell operates only on one time step and predicts another unique time step (T=1 and F=1). DEEP-AR predicts the mean and standard deviations of the forecast value, enabling the model to exhibit stochastic behavior and to learn the uncertainty on the forecast.

NBeats [3] directly operates on all prior times $X_{1:T}$ and uses a hierarchical RNN structure to capture trends, seasonality, and repetitions, resulting in a hidden representation Z. From this time compression, the model can reproduce the prior TS and forecast multiple time steps ahead $X_{T+1:T+F}$. The hierarchical structure allows for interpreting the time series.

Transformers [19] encode the given TS with multiple self-attention layers in order to capture repetitions and logic between time steps. Each time step is input into its own self-attention layer. Once the time dimension is effectively compressed into Z, the model decodes the compressed representation to generate one (or multiple) time step forecast(s) $X_{T+1:T+F}$, depending on the model.

### 2.2. Compression by Source Masking

According to Tishby’s original IB formulation, the downstream task was a classification. The form of information minimization in the IB is not necessary via the dimension reduction or the addition of noise, but it can be via any lossy operation, such as lossy compression or masking. We propose to address the IB principle for MTS via source masking, dimension reduction, and prediction of the masked parts. Using masks, Equation (4) can be rewritten as:

(8) $$L\left(\Theta ,\Phi \right)=I_{\Theta }\left(X_{1:T+F}⊙M_{1:T};Z_{ib\_tr}\right)-\beta I_{\Phi }\left(Z_{ib\_tr};X_{1:T+F}⊙M_{T+1:T+F}\right),$$

where ⊙ is the element-wise product, also known as the Hadamard dot product, where $M_{1:T}$ and $M_{T+1:T+F}$ are binary time masks that have ones at the indexed time positions, 1:*T* or T+1:T+F, and zeros at the other time positions. Note that Equation (8) is very close to the formulation of IB for AEs in Equation (1) but with additional masks. Without masks, the bottleneck not only holds statistics for the transitions 1:T→T+1:T+F, but also contains all statistics for the reconstruction of the entire sequence 1:T+F. In that case, the bottleneck is reduced to an AE bottleneck $Z_{ib\_ae}$ with dimension reduction purposes only. In contrast, with masking, the bottleneck $Z_{ib\_tr}$ is designed to learn transition statistics 1:T→T+1:T+F.

### 2.3. Compressing Multi-Dimensional Data by Extreme Spatiotemporal Dimension Reduction

Previous subsections have not specifically taken into account the multi-dimensional aspects of MTS. Instead of scalar values $X_{t}$ for TS, each time step is a vector $X_{t}=\left[X_{t}^{1},\ldots ,X_{t}^{M}\right]$ or tensor for MTS, usually referred to as the *spatial dimension*. Previous works [59] show that models designed for TS usually fail to capture the dependencies between spatial and temporal dimensions. This difficulty has been addressed in two ways: at each time step, adding a model for the spatial interdependencies [60], or designing the spatiotemporal interdependencies jointly [18]. The first proposition models the joint spatial distributions $p(X_{t}^{1},\ldots ,X_{t}^{M})$ for each *t*, either explicitly with GNN [21] or implicitly with the spatial compression [1]. The second proposition models the spatiotemporal joint distribution $p(X_{1}^{1},\ldots ,X_{1}^{M},\ldots \ldots ,X_{T}^{1},\ldots ,X_{T}^{M})$ with joint spatiotemporal attention [18], as well as an encoder–decoder structure made of attention layers for each spatiotemporal scalar variable. The latter performs better than the first one because the spatiotemporal dependencies $X_{t^{\prime }}^{m^{\prime }}|X_{t}^{m}$ are explicitly designed, whereas the first type decomposes spatiotemporal dependencies in two steps: spatial plus temporal $X_{t}^{m}\to X_{t}^{m^{\prime }}\to X_{t^{\prime }}^{m^{\prime }}$ or temporal plus spatial $X_{t}^{m}\to X_{t^{\prime }}^{m}\to X_{t^{\prime }}^{m^{\prime }}$.

We propose to handle the spatiotemporal compression of the masked MTS data $X_{1:T+F}⊙M_{1:T}$ present in Equation (8) using successive two-dimensional (temporal and spatial)-strided partial convolutions PConv [4]. When the stride is 2, each PConv layer divides the 2 spatiotemporal dimensions by 2, such that, with an adequate number of hidden PConv layers, the spatiotemporal dimensions of the bottleneck $Z_{ib\_tr}$ are reduced to 1×1, and the resulting mask $M_{ib\_tr}$ is a 1×1 unit matrix. This means that $Z_{ib\_tr}⊙M_{ib\_tr}=Z_{ib\_tr}$ and the posterior $X_{1:T+F}⊙M_{T+1:T+F}=X_{T+1:T+F}$ can be decoded from $Z_{ib\_tr}$ without any masking considerations. Finally, our approach proposes to assimilate the MTS forecasting problem as an image extension problem, where MTS, being two-dimensional, can be visualized as pseudo-images and processed with classical CV layers as convolutions.

Convolutions also present non-negligible advantages because they locally model spatiotemporal dependencies present in these MTS pseudo-images. Moreover, because of the spatiotemporal compression structure, each successive strided PConv hidden layer creates a more global model of the spatiotemporal dependencies, such that, in the end, the bottleneck, $Z_{ib\_tr}$ fully models the global spatiotemporal dependencies.

### 2.4. Performing the Forecast

#### 2.4.1. Decoder

Section 2.3 shows that the IB for MTS forecasting is equivalent to a pseudo-image extension problem, where the masked source can be compressed with successive PConv layers. A very efficient image inpainting model that also uses masks and PConv layers is proposed in [4]. Instead of simply decoding the masked posterior from the bottleneck, it proposes to use a U-Net structure with partial convolution in order to output the full image with the unmasked parts reconstructed and the masked parts predicted. We propose designing the decoder in the same way, i.e., using skipping layers and a structure similar to the encoder, consisting of PConv hidden layers, such that the global model is a U-Net architecture with successive PConv layers that extends to the bottleneck, followed by successive partial deconvolution layers PDConv [4]. The information flow of such a model is sketched by the following Markov chain: 
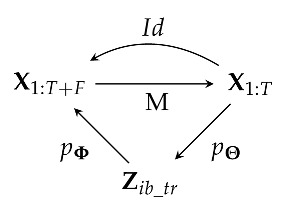
 For this configuration, and under a Laplacian assumption for the distribution $p_{\Phi }\left(X_{T+1:T+F}|Z_{ib\_tr}\right)$, the third term of the IB loss $L_{3}\left(\Theta ,\Phi \right)$ in Equation (8) becomes equivalent to:

(9) $$L_{3}^{Lap,UNet}\left(\Theta ,\Phi \right)=E_{p_{D}\left(X_{1:T+F}\right)}\left[E_{p_{\Theta }\left(Z_{ib\_tr}|X_{1:T+F}⊙M_{1:T}\right)}\left[{∥X_{1:T+F}-{\tilde{X}}_{1:T+F}∥}_{1}\right]\right],$$

where ${\tilde{X}}_{1:T+F}$ is the output of the U-Net. This equivalence and more details are given in Proof of Equation (A13) and Remark A1 of the Appendix A.

#### 2.4.2. Partial IB Loss with U-Net

In Section 2.1, we show that for MTS, the IB principle imposes the three losses defined in Equation (6). Moreover, if one assumes a Laplacian distribution for $p_{\Phi }\left(X_{T+1:T+F}|Z_{ib\_tr}\right)$, Equation (9) shows that the cross-entropy $H(p_{\Theta },p_{\Phi })$, which is the third part, $L_{3}$, of the upper bound on the IB loss, can be reduced to $L_{3}^{Lap,UNet}$, an average Manhattan distance, which is also referred to as MAE between the output ${\tilde{X}}_{T+1:T+F}$ and the genuine $X_{T+1:T+F}$, when one samples $Z_{ib\_tr}$ from the prior samples $X_{1:T}$ of the training data, assuming that the Laplacian distribution of $p_{\Phi }\left(X_{T+1:T+F}|Z_{ib\_tr}\right)$ is too restrictive for real MTS datasets, and a simple MAE cannot provide the best forecasting performance.

Image extension and inpainting processing works [4,23,61] use the same design for their models. They develop powerful image inpainting models, which involve recovering masked or missing parts of images, not only in central regions but also on the borders. These models use U-Net [31] with gated, partial, or dilated convolutions and complex losses based on the Manhattan distance MAE, such as style loss by the Gram matrix computation [62], perceptual loss by the VGG-16 hidden layer value computation [29], and/or adversarial loss [63]. One of the most efficient of these works [4] decomposes the loss into six partial losses, each of which is responsible for optimizing specific errors between the global genuine $X_{1:T+F}$ and the output ${\tilde{X}}_{1:T+F}$. The rest of this section will provide interpretations of these losses in the context of MTS. The first partial loss, $L_{valid}$, is related to the *valid* or *prior* parts of the pseudo-images that can be easily reconstructed with the skipping layers:

(10) $$L_{valid}=\frac{1}{N_{pi}}∥M_{1:T}⊙\left({\tilde{X}}_{1:T+F}-X_{1:T+F}\right){∥}_{1}=\frac{1}{N_{pi}}{∥{\tilde{X}}_{1:T}-X_{1:T}∥}_{1},$$

where $N_{pi}=M\times \left(T+F\right)$ is the size of the MTS pseudo-image $X_{1:T+F}$ or the number of pixels. As explained in Section 2.4.1 and Proof of Equation (A13) from the Appendix A, this part of the loss is responsible for the equivalence between the partial IB loss $L_{3}$ defined in Equation (6) and $L_{3}^{Lap,UNet}$, which is the Manhattan distance MAE between the U-Net output ${\tilde{X}}_{1:T+F}$ and the genuine $X_{1:T+F}$ due to the presence of the skipping layers. It ensures an easy reconstruction of the known parts, and forces the bottleneck $Z_{ib\_tr}$ to learn the transition statistics 1:T→T+1:T+F but not the reconstruction statistics 1:T→1:*T*. As a consequence, in some way, it also softly minimizes $L_{1}\left(\Theta \right)=H_{p_{\Theta }}\left(Z_{ib\_tr}\right)$, but without forcing this loss to reach a local minimum. The second loss is noted $L_{hole}$ for the masked parts of the pseud-images that need to be forecasted:

(11) $$L_{hole}=\frac{1}{N_{pi}}∥\left(1-M_{1:T}\right)⊙\left({\tilde{X}}_{1:T+F}-X_{1:T+F}\right){∥}_{1}=\frac{1}{N_{pi}}{∥{\tilde{X}}_{T+1:T+F}-X_{T+1:T+F}∥}_{1}.$$

This loss is exactly the IB partial loss $L_{3}=L_{hole}$ of Equation (6) if we assume $p_{\Phi }\left(X_{T+1:T+F}|Z_{ib\_tr}\right)$ to follow a Laplacian distribution. Under this assumption, this loss forces the encoder to actually let $Z_{ib\_tr}$ represent the transition statistics between the prior and the posterior; this loss also lets the decoder generate well the posterior from this bottleneck representation. It is interesting to note the $L_{valid}+L_{hole}=L_{3}^{Lap,UNet}=L_{3}$ under the Laplacian assumption of $p_{\Phi }\left(X_{T+1:T+F}|Z_{ib\_tr}\right)$ and for the U-Net flow of information. The three next losses are also MAE, but instead of being directly computed on the genuine X and prior $\tilde{X}$, they are computed on deep representations of these variables. These three losses are referred to as *perceptual* and *style* losses:

(12) $$\begin{array}{cc} L_{perceptual} & =2\sum_{p\in H_{VGG}}\frac{∥\Psi_{p}\left({\tilde{X}}_{T+1:T+F}\right)-\Psi_{p}\left(X_{T+1:T+F}\right){∥}_{1}}{N_{\Psi_{p}\left(X_{1:T+F}\right)}}\\ \; & \;+\sum_{p\in H_{VGG}}\frac{∥\Psi_{p}\left({\tilde{X}}_{1:T}\right)-\Psi_{p}\left(X_{1:T}\right){∥}_{1}}{N_{\Psi_{p}\left(X_{1:T+F}\right)}},\\ L_{style_{out}} & =\sum_{p\in H_{VGG}}\frac{∥\Gamma_{p}\left({\tilde{X}}_{1:T+F}\right)-\Gamma_{p}\left(X_{1:T+F}\right){∥}_{1}}{N_{\Psi_{p}\left(X_{1:T+F}\right)}},\\ L_{style_{comp}} & =\sum_{p\in H_{VGG}}\frac{∥\Gamma_{p}\left(\left[X_{1:T},{\tilde{X}}_{T+1:T+F}\right]\right)-\Gamma_{p}\left(X_{1:T+F}\right){∥}_{1}}{N_{\Psi_{p}\left(X_{1:T+F}\right)}}, \end{array}$$

where $\Psi_{p}$ are selected hidden layers of a pre-trained VGG-16 [64] deep image model classifier, where $H_{VGG}$ is the set of selected hidden layer indices, and $\Gamma_{p}\left(X\right)$ are Gram operators on the hidden layers of VGG [29], as defined by $flatten\left[\Psi_{p}\left(X\right)\right]\cdot flatten{\left[\Psi_{p}\left(X\right)\right]}^{T}$. While the MAE of $L_{3}^{Lap,Unet}$ is equivalent to the IB partial loss $L_{3}$ from Equation (6) with the Laplacian assumption of $p_{\Phi }\left(X_{T+1:T+F}|Z_{ib\_tr}\right)$, for these losses, MAE is applied to deep hidden representations of X and $\tilde{X}$. In a similar manner to the normalizing flow [65], where each hidden layer of a deep network modifies the distribution, hidden layers of the VGG and Gram operators are deterministic mappers that modify the distribution of $X_{T+1:T+F}$. As a consequence, we assume that the MAEs of $L_{perceptual}$ and $L_{style}$ can provide equivalents to the IB partial loss $L_{3}$ from Equation (6) for other distributions $p_{\Phi }\left(X_{T+1:T+F}|Z_{ib\_tr}\right)$ than the simple Laplacian. Hidden layers of the VGG-16 model capture the statistics related to the prediction of the humanly recognizable class to which an image may belong. The losses using MAE on these hidden layers are assumed to measure human perceptual features, and Gram operators are known to capture styles in an image [62]. Finally, a combination of $L_{valid}$, $L_{hole}$, $L_{perceptual}$, $L_{style_{out}}$, and $L_{style_{comp}}$ provides an equivalent to the IB partial loss $L_{3}$ for a less restrictive assumption than the simple Laplacian distribution of $p_{\Phi }\left(X_{T+1:T+F}|Z_{ib\_tr}\right)$. The last loss is referred to as *total variation*:

(13) $$L_{tv}={∥X_{T}-{\tilde{X}}_{T+1}∥}_{1}.$$

In image extension or inpainting problems, this loss forces the borders of the predicted holes to be smooth, which is a valid assumption for large images with high definition. In the context of MTS, this loss forces the first forecasted time step T+1 to be similar to the last known time step *T*. This smoothness assumption is also valid for the majority of real-world observations, where most of the functions are continuous.

The U-Net structure does not impose specific distributions for $p(Z_{ib\_tr})$ and $p(Z_{ib\_tr}|X_{1:T})$, such that the partial IB losses $L_{1}$ and $L_{2}$ are intractable, but U-Net imposes an extreme spatiotemporal compression in the bottleneck $Z_{ib\_tr}$. The source masking, combined with U-Net’s skipping layers, allows for a limitation of $L_{1}$, as the transition statistics 1:T→T+1:T+F rather than the reconstruction ones 1:T→1:T are learned. For these reasons, the loss used for the model in the experiments is the following partial IB loss:

(14) $$\begin{array}{c} L_{total}=L_{valid}+a_{1}L_{hole}+a_{2}L_{perceptual}+a_{3}\left(L_{style_{out}}+L_{style_{comp}}\right)+a_{4}L_{tv}, \end{array}$$

where $a_{1}$, $a_{2}$, $a_{3}$, and $a_{4}$ are empirically defined hyperparameters. In practice, we use $a_{1}=6$, $a_{2}=0.05$, $a_{3}=120$, and $a_{4}=0.1$, and only the hidden layer activations $\Psi_{p}$ with indices p=3,6, and 10 of the VGG-16 [64] are used to compute $L_{style_{out}}$, $L_{style_{comp}}$, and $L_{perceptual}$, such that $H_{VGG}=\left\{3,6,10\right\}$, such as in [4]. These parameters were fine-tuned in [4] for image datasets, where pixels take values between 0 and 1. The pseudo-images created from the IRIS, AL, and PB datasets also have pixels ranging from 0 to 1. Because of the good results we achieved with these parameters, we assumed that it was enough to prove the efficiency and adaptability of the described method on different types of MTS data, and we did not attempt to further fine-tune these hyperparameters for each dataset evaluated.

#### 2.4.3. IB Interpretation with the Partial Loss

The encoder has a mapping form that consists of two parts. The first encoding corresponds to the masking, i.e., vector $X_{1:T+F}$, only $X_{1:T}$ is retained as the input to the second part. Thus, in principle, the masking part can be any stochastic map that masks the parts to be predicted. This technique is similar to recent methods referred to as *masked image modeling* (MIM) [66,67] and it is often used in the pretraining of image autoencoders or transformers [66,68]. The second encoding part is a nonlinear embedding implemented as a deterministic encoder, which is the compression part of the U-Net, along with its connecting layers. This second compression is guided by successive masked convolutions with strides of the order of 2 to obtain a bottleneck by the dimension reduction of shape 1×1×K, i.e., where the spatial and temporal dimensions are reduced to 1. This compressed representation is noted as $Z_{ib\_tr}$ and referred to as the bottleneck in the paper. The masking, together with the deterministic nonlinear embedding, form a stochastic mapping, and can be considered as the equivalent part of the stochastic encoder in the IB framework.

In the end, a nonlinear decompression implemented in a form of a deterministic decoder predicts $X_{1:T+F}$ from this bottleneck representation $Z_{ib\_tr}$ and from the skipping layers that map the prior $X_{1:T}$ information between the input and output. Theoretically, in Section 2.3, we show that the bottleneck should only retain the necessary information of the transition statistics 1:T→T+1:T+F between the prior and the posterior, and not the statistics of the reconstruction of the prior $X_{1:T}$. This is because all of the information from the prior $X_{1:T}$ is transmitted to the output via the skipping layers. This shortcut flow of information is specific to the structure of U-Net, is performed without compression, and preserves the spatiotemporal positions of the prior information. As such, theoretically, only the statistics of the transitions 1:T→T+1:T+F are retained in the bottleneck $Z_{ib\_tr}$ and the following Markov chain holds:

(15) $$X_{1:T}\to Z_{ib\_tr}\to X_{T+1:T+F}.$$

Moreover, to better understand the role of the bottleneck, we can consider these two thought experiments:

- If we remove the skipping layers, the bottleneck should not only retain the statistics of the transitions from the prior to the posterior but also the reconstruction statistics of the prior.
- If we also remove the source masking of the posterior in $X_{1:T+F}$, the model is reduced to an autoencoder (AE) and the bottleneck is supposed to perform a dimension reduction of the MTS. Because of the curse of dimensionality, this technique is commonly used to further perform better classifications on the bottleneck representation than on the raw high-dimensional MTS data.

In our case, instead of imposing a distribution of the latent space, such as for VAE, we apply special masking jointly with a dimensionality reduction. This framework can be considered as the lossy part of the information encoding.

### 2.5. Proposed Model

The model designed in Section 2.4 corresponds to a traditional U-Net complemented with masks and partial convolutions [4,31]. Multidimensional successive PConv and PDConv layers with a stride of 2 are used when the data are MTS (M>1). The bottleneck must have a 1×1 spatiotemporal shape; because of stride 2, the input pseudo-images must be zero-padded to become squares with a power of 2. As a consequence, if $2^{l}\times 2^{l}$ is the spatiotemporal shape of the pseudo-image, the designed U-Net must have *l* successive PConv followed by *l* successive PDConv. Training is performed with 100 epochs and the Adam optimization of gradient descent with a $2\times 10^{-4}$ learning rate for the loss defined in Equation (14). The model could be generalized to N-dimensional PConv and PDConv layers when several dimensions are necessary to model each time step. For instance, in videos, each time step is an image with horizontal and vertical spatial dependencies.

**Figure 2:**
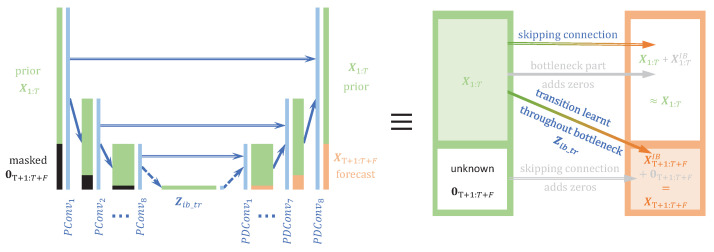
Schematic analogy between the IB principle and image extension: (**Left**) schematically shows the time prediction under the IB principle, with compression and decoding, using PConv and DPConv and skipping connections to form a variant of U-Net. (**Right**) is an equivalent representation seen as the image extension, where the skipping layers connect $X_{1:T}$ from the input to the output, and the bottleneck principle allows predicting $X_{T+1:T+F}$ from $X_{1:T}$.

**Example of architecture when 128<max(M,T+F)≤256:** The maximum spatiotemporal size is max(M,T+F), which can also be interpreted as the maximum width or height of the pseudo-images. In this situation, input pseudo-images are zero-padded to obtain a square shape 256×256, and the investigated model is sketched in Figure 2; it uses the classical image extension architecture, U-Net [31], which has a symmetrical structure made of an encoder and a decoder, both with 8 layers. Implementation details of the architecture are given in the Appendix B, Table A2. The encoded representation $Z_{ib\_tr}$ has a size of 1×1×512. Each layer of the encoding part divides the width and height with strides of a factor of 2, and increases the number of channels up to 512. The decoder has a symmetrical structure, but the inputs of each layer are concatenations of upsampled versions of the previous layer’s outputs with the output of the symmetrical encoding layer. It is combined with partial convolutions (PCs) that were proposed in [4] to handle the masked data. These convolutions are applied at each hidden step and are designed to not take into account the missing data, such that $X_{T+1:T+F}$ from $X_{1:T+F}$ at the input layer. At each step of the encoding, the proportion of the masked part is reduced. Each PC is followed by a batch normalization and a ReLU activation, but for the last output layer, the activation is a sigmoid. For the training, we used input images of size 240×240, which were center-padded by zeros to make an image of size 256×256 for fitting the U-Net input size. Because of the 2 strides at the encoding steps and the 1×1 size of the latent representation, a U-Net with 8 encoding layers requires input sizes of $2^{8}=256$.

When the input of the maximum spatiotemporal size max(M,T+F) is smaller than 128, the model needs less than 8 PConv and PDConv layers for the encoder and decoder parts of the U-Net. In general, the number of layers must be $\log_{2}\left(\max\left(M,T+F\right)\right)$ to ensure a 1×1 spatiotemporal size in the bottleneck $Z_{ib\_tr}$.

In [4], they specify that because of the masks, batch normalization prevents training from converging. This is because the mean and standard deviation values of batch normalization layers for each sample are biased by the masks. As a consequence, they train the first half of epochs with trainable batch normalization layers, and they freeze the batch normalization layers for the second half of epochs. This is done in TensorFlow by setting the layer parameter trainable=False during training. In our case, all sample masks have the same size and spatiotemporal positions. As a consequence, the mean and standard variations of the samples are not very biased by those masks. Actually, the experimentation showed that freezing the batch normalization for the last epochs did not lead to improved performance.

### 2.6. IRIS Dataset

**Figure 3:**
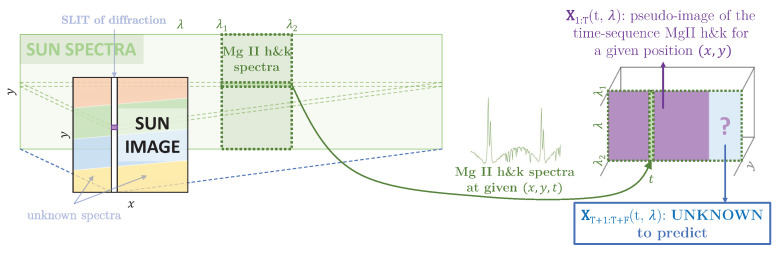
Problem formulation: (x,y) represent the spatial coordinates, λ and *t*, respectively, represent the spectral and time coordinates. NASA’s IRIS satellite integrates a mirror from which the *Sun image* or videos are captured by a sensor paired with a wavelength filter chosen among 1330Å, 1400Å, 2796Å, and 2832Å. This mirror holds a vertical slit from which the diffraction occurs. The *x* position of the slit can vary in time and is chosen before the observation. A sensor behind the mirror captures the *Sun spectra* for each vertical position *y* of the *Sun’s image*, but only at the *x* position of the slit. We only consider the MgIIh/k data, which are between $\lambda_{1}=2793.8401\;Å$ and $\lambda_{2}=2806.02\;Å$, and we consider all available time sequences.

IRIS is NASA’s interface region imaging spectrograph satellite [69]. IRIS observes regions of the atmosphere of the Sun with many different settings of possible observations recorded in a specified cadence. A time sequence is encoded into a two-dimensional representation in the form of images. We used the designed model to predict time sequence data provided by the IRIS mission. The basis of the IRIS satellite data retrieval is shown in Figure 3. Each observed event is composed of a maximum of four videos of a selected region on the surface of the Sun, together with spectral videos, where a slit is positioned to perform the diffraction [70].

In this work, only the spectral data from *MgII h*&*k* lines, between 2793.8401Å and 2806.02Å, were considered. This wavelength’s range is represented by a vector of size 240. According to modern solar physics theories, spectral data are supposed to contain most of the information on the physics of the Sun, and *MgII h*&*k* lines are considered some of the best lines to recover information from the chromosphere [71]. The predictions of solar spectral data are crucial for different reasons, including the solar flares forecasting and solar activity in general.

IRIS data are publicly available (iris.lmsal.com/data.html) (accessed on 20 February 2023) but only part of the data is labeled [72]. Only three types of solar activities were considered for this study: quiet Sun (QS), where nothing special appears, active region (AR), where some activity is observed, such as solar prominence, filaments, jets, and flaring profile (FL), when a flare appears during the observed event. Each event is assigned a hierarchical label, such that an event is labeled FL even when it includes AR time steps. The data are normalized at each time step by its maximum value, such that the maximum value at each time step, or the *intensity* of the signal, is reduced to one. This allows for an easier comparison of spectral profiles at each time step, and simplifies the process in terms of the ML.

#### 2.6.1. Problem Formulation

**The IRIS restrictions of online observations:**Figure 3 explains the IRIS observations in the atmosphere of the Sun with images of given wavelengths and spectra of given positions. Despite the very high precision of IRIS and its capacity to observe a very wide range of astrophysical parameters in time and space, significant difficulties inherent to online observations remain. Spectral observations are limited in time and space as they only correspond to the position of the slit at a given time, which may vary, and the satellite has to store the data before sending them to Earth-based stations [70]. IRIS observations are, therefore, very sparse in all of the potential observable parameters and they may lack a lot of data from other spatial positions. We may also be interested in further observations after the termination of the acquisition/recording session limited by the IRIS storage memory capacity.

Spectral time sequence forecasting represents a significant step forward in flare forecasting and assists in planning satellite observations. Figure 3 presents the solar–physical interpretation of the spectral time sequence data, represented as images $X_{1:T+F}^{1:M}$ of physical dimensions time×wavelength with 1≤timet≤T+F, 1≤wavelengthλ≤M; the left part $X_{1:T}^{1:M}$ (1≤t≤T, 1≤λ≤M) corresponds to the known prior sequence, and the right part, $X_{T+1:T+F}^{1:M}$ (T+1≤t≤T+F, 1≤λ≤M), masked, predicted, or genuine, is the sequence that has to be predicted by the model.

**Figure 4:**
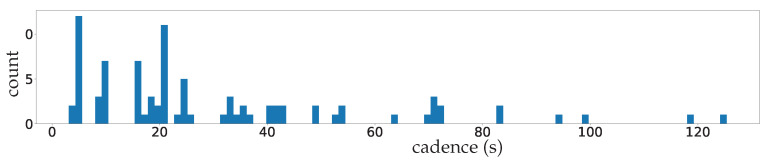
Histogram of the cadences in seconds/time steps.

**Non-homogeneous cadences of the data** time series modeling are usually performed by RNNs [73] or LSTMs [6], as briefly summarized in Figure 1. These models are designed for time series with fixed given cadences; Figure 4 shows the wide variety of our data cadences, making the use of RNN or LSTM difficult. To represent the time sequences $X_{1:T+F}$ of the data under a common cadence, one should represent them by a cadence equal to the greatest common divisor of all of the cadences, which would obviously make those time sequences $X_{1:T+F}$ highly sparse and penalize the learning of transitions between time steps.

**Clustering spectral data:** The 53 clusters of MgIIh/k lines found in [55] allow interpreting the physics on the surface of the Sun. We can compare the original and predicted time sequences through their clustered time sequences in order to prove the utility of our forecasting model in solar–physics by conserving the types of activities.

**Astrophysical features:** In [72], the authors defined ten solar spectra features to be used as dimensional reductions of spectral data for activity classification purposes. We studied the conservation of these features in the forecasted sequences to show the applicability to astrophysics.

#### 2.6.2. Proposed Approach

As described in Section 2.6.1, a solar spectral time sequence is represented by an image $X_{1:T}$ and the model has to predict the continuation, which is the time sequence equivalent to the image $X_{T+1:T+F}$. $X_{T+1:T+F}$ should be the right extension of the image $X_{1:T}$, where each column of the image represents a spectrum at a growing time step from the left to the right. Because of the architecture of the image extension models, the input and output images have the same dimensions. The targeted output image $X_{T+1:T+F}$ is the concatenation of $X_{1:T}$ with $X_{T+1:T+F}$ on the right of it, whereas the input image $Concat(\left[X_{1:T},0_{T+1:T:F}\right])$, has a blank image $0_{mask}$ of the same shape as $X_{T+1:T+F}$ on the right of $X_{1:T}$.

For both recurrent and image extension models, the input has the same information, organized differently, and the output of the image extension models differs by the left concatenation of the input. Figure 2 shows the skipping layers in the image extension models that help the transition of $X_{1:T}$ from the input to the output.

### 2.7. Other MTS Dataset

Two other datasets are used to provide a baseline evaluation between our IB-designed models and concurrent ones.

- **AL** dataset: The solar power dataset for the year 2006 in Alabama is publicly available (www.nrel.gov/grid/solar-power-data.html, accessed on 20 February 2023). It contains solar power data for 137 solar photovoltaic power plants. Power was sampled every 5 min in the year 2006. Preprocessing was conducted to only extract daily events by ignoring nights when data were zero. At each 5-min interval, the data consisted of vectors with 137 dimensions, and these vectors were normalized by their maximum coordinates. For example, in the case of IRIS data, the maximum value at each time was always set to 1.
- **PB** dataset: PeMS-BAY data [74] are publicly available (https://zenodo.org/record/5146275#.Y5hF7nbMI2w, accessed on 20 February 2023) and were selected from 325 sensors in the Bay Area of San Francisco by the California State Transportation Agency’s Performance Measurement System [75]. The data represent 6 months of traffic speeds ranging from January 1 to May 31 2017. At each 5-minute interval, the data consist of vectors with 325 dimensions, and these vectors are normalized by their maximum coordinates. For example, in the case of IRIS data, the maximum value at each time was always set to 1.

### 2.8. Complementary Classifiers to Show Consistency with Applied Sciences

The experimental proof was conducted on IRIS-labeled data to show that the proposed model is not simply capable of predicting possible images but also capable of predicting the information logic behind them. It is common for MTS data (that are to be forecasted) to be classified based on types of activity. There are multiple examples of MTS types of activity, including displacement, boom, euphoria, profit-taking, and panic classifications. In astrophysics, when dealing with solar observations, the types of activity can be categorized as quiet, active, and flaring.

We implemented a classifier composed of eight strided convolutional layers, with dense ending layers, which allowed it to output a vector of size corresponding to the number of classes. This classifier was trained on labeled MTS data ${\left\{\left(X_{1:T+F},K\right)\right\}}_{D}$, where K stands for the categorical one-hot vector representing the class activity for the series $X_{1:T}$. Once trained, the classifier was used to classify the prior, the genuine, and the predicted forecasts. The classification accuracy between the genuine and the predicted forecasts was evaluated together with the true skill statistic (TSS), which is also known as the Hansen and Kuiper skill score [76], and the Heidke skill score (HSS) [77], which is also known as *kappa* [78]. These two scores were evaluated globally and for each class of prediction. For one class, these scores are defined as follows:

(16) $$\begin{array}{c} TSS=\frac{tP\times tN-fP\times fN}{gP\times gN},\;\mathrm{and}\;HSS=2\frac{tP\times tN-fP\times fN}{gP\times pN+gN\times tP}, \end{array}$$

where *t* stands for *true*, *f* is *false*, *g* is *genuine*, *p* is *predicted*, *P* represents *positives*, and *N* represents *negatives*. In a classification with more than two classes, [79] shows that these scores can be defined by generalization, as follows:

(17) $$\begin{array}{c} TSS=\frac{trace(CM-ICM)}{trace(CM^{*}-ICM^{*})},\;\mathrm{and}\;HSS=2\frac{trace(CM-ICM)}{trace(CM^{*}-ICM)}, \end{array}$$

where trace(.) is the diagonal sum operator for a matrix of dimension m×m, which is eventually larger than 2×2. $CM={\left(Count_{g_{i},p_{j}}\right)}_{i,j}$ is the confusion matrix holding the joint counts of genuine classification cases $g_{i}$ (rows) versus forecast classification cases $p_{j}$ (columns), and $ICM={\left(\frac{Count_{g_{i}}\times Count_{p_{j}}}{total\;count}\right)}_{i,j}$ is the confusion matrix expected when the genuine and forecast classifications are independent events. $CM^{*}=diag\left(Count_{g_{i}}\right)$ is the expected diagonal confusion matrix when the classifications are ideal (an ideal classification is defined by $Count_{g_{i},p_{j}}=0\;\mathrm{for}\;\mathrm{all}\;i\neq j$, such that the confusion matrix CM is diagonal); $ICM^{*}$ is the corresponding expected confusion matrix when genuine and forecast classifications are independent events.

### 2.9. Comparison with Other Models

To our knowledge, for almost all MTS datasets, current state-of-the-art (SOTA) datasets are achieved by TS decomposition networks, such as NBeats [3] and SCINet [80], and by pre-trained models designed as graph neural networks [60,81] or transformers [17]. We compare our proposed IB-MTS model with three types of models:

- Multiple successive IBs, where each time step is an instance of the IB formulation. This includes multidimensional RNN, LSTM, and GRU models [5,6,15].
- Composition of two successive IBs: A spatial IB is followed by a temporal IB. For example, this would involve encoder–decoder models using successive RNN, LSTM, or GRU recurrent models at each layer of compression and decoding [82,83].
- Unique joint spatiotemporal IB: The encoder jointly compresses spatial and temporal dimensions of the prior into a bottleneck with an extreme spatiotemporal dimensional reduction; this is our proposed IB-MTS formulation.
- MTS decomposition model, such as NBeats [3].

**Figure 5:**
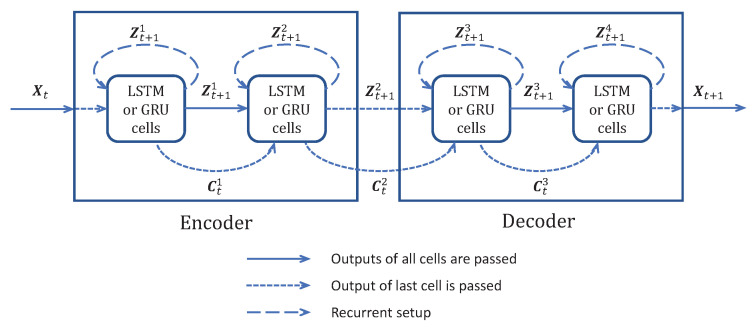
Structure of the ED-LSTM and ED-GRU models used for comparison. $C_{t}^{i}$ represents the hidden state vectors for GRU cells, combined with cell state vectors for LSTM cells.

Names and details of the concurrent evaluated models are listed below:

- **LSTM** model: An LSTM cell [6] performs the one-step-ahead forecast and is trained to predict $X_{t+1}$ from $X_{t}$. It incorporates one layer with *M* LSTM units. For instance, for the 240×240 spatiotemporal dimensions of IRIS data, the 180 first time steps are the prior data, and the 60 last time steps are the posterior data to forecast. This model is designed with 240 spatial LSTM/GRU units looped 180 times and all of the cell outputs are returned by the model using TensorFlow option return_sequences=True. This layer returns a 180×240 output and only the last 60 time steps are kept. Moreover, a source masking of the posterior is applied to the input and an identity skipping layer is added to transmit the prior $X_{1:T}$ to the output at the same temporal positions in $X_{1:T+F}$, such that the LSTM layer only accounts for predicting the posterior part $X_{T+1:T+F}$. The number of units is directly determined by the shape of the input and output data. Details of the architecture are given in the Appendix B, Table A3.
- **GRU** model: A GRU [15] cell is trained to predict $X_{t+F}$ from $X_{t}$. The structure and number of units are the same, similar to the LSTM models, but GRU cells are used instead of LSTM ones. Details of the architecture are given in the Appendix B, Table A3.
- **ED-LSTM** model: A version using LSTM cells with an encoder and a decoder was implemented as described in Figure 5. Because of the encoder and decoder structures, we name it ED-LSTM. This model conducts multiple step-ahead forecasts and can forecast $X_{T+1:T+F}$ from $X_{1:T}$. The model incorporates LSTM cells organized into four layers: two layers of encoding into a bottleneck and two layers of decoding from the bottleneck. The first encoding layer is composed of 100 spatial units looped 180 times on the prior IRIS data and all of the cell outputs are returned by the model using TensorFlow option return_sequences=True, returning a 180×100 spatiotemporal output accounting for a spatial compression. The second encoding layer is composed of 100 spatial units looped 180 times and only the last cell outputs of the recurrences are returned, returning a 100-dimensional bottleneck that accounts for a spatial compression followed by a temporal compression. This bottleneck representation is repeated 60 times for IRIS data in order to model the decoding of 60 posterior time steps to forecast. After this repetition, the data are 60×100 and fed to the first decoding layer with 100 spatial units looped 60 times and initialized with the states obtained from the second encoding layer; indeed, the structure is symmetrical, such that the first and second decoding layers are, respectively, the images of the second and the first encoding layers. All cell outputs are returned by the model using TensorFlow option return_sequences=True, such that a 60×100 spatiotemporal output is returned. The second layer of the decoder is designed with 100 spatial units looped 60 times and initialized with the states obtained from the first encoding layer, such that a 60×100 output is returned. In the end, a time-distributed dense layer is used to map the 60×100 output data into a 60×180 MTS data format. For these models, the input and output shapes are determined by the data and one can only change the number of spatial cells $n_{1}$ and $n_{2}$ used, respectively, in the first and second layers of the encoding part. $n_{2}$ determines the dimension of the bottleneck and on the IRIS data, $180>n_{1}\geq n_{2}\geq 1$. Our experiments show that the results of these models do not depend much on the values of $n_{1}$ and $n_{2}$, but significantly drop when $n_{2}$ is very small, close to 1. Details of the architecture are given in the Appendix B, Table A4.
- **ED-GRU** model: This model follows the same structure as the ED-LSTM but with GRU cells instead of LSTM cells. The structure and number of units are the same as with ED-LSTM models, but GRU cells are used instead of LSTM ones. Details of the architecture are given in the Appendix B, Table A4.
- **NBeats** model: We use the code given in the original paper [3]. This model can forecast $X_{T+1:T+F}$ from $X_{1:T}$. The model is used in its generic architecture as described in [3], with 2 blocks per stack, theta dimensions of (4,4), shared weights in stacks, and 100 hidden layers units. For IRIS data, the prior is 180×240, the forecast posterior is 60×240, and the backcast posterior is 180×240, but we also use a skipping layer to connect the input to the backcast posterior, such that the model is forced to learn the transition between the prior and the forecast posterior. we attempted NBeats with other settings and other numbers of stacks without the gain of performance, and when the number of stacks became greater than 4, the model failed to initialize on our machines.

Table 1 provides the number of parameters for each model. The evident drawback of our proposed IB-MTS is the high amount of parameters inherent to U-Net structures with several levels of deepness. The number of parameters for the concurrent evaluated models is determined by the shape of the input data or by hardware constraints for NBeats. We believe that our model also integrates more parameters because it is based on a jointly spatiotemporal compression whereas the others are based either on a temporal succession of spatial IB or on a combination of a spatial followed by a temporal IB. In theory, the number of parameters for our proposed model should be the square of the number of parameters of the models based on the *N* successive spatial IB. In practice, it is less than the square, and still able to model a joint distribution, which is not the case for the other ones.

Moreover, a remarkable thing is that our proposed IB-MTS model needs less time to be trained than the others, except for simple RNNs. The differences in duration are even more significant compared to NBeats. NBeats has a very small amount of parameters but is very slow to train compared to the other models. From our experience, instantiating the NBeats model can be quite slow, and in practice, it is impossible to instantiate with much more layers and parameters than the vanilla one. For instance, 120 GB of RAM was not enough when we attempted to set the NBeats model with millions of parameters.

## 3. Results

**Figure 6:**
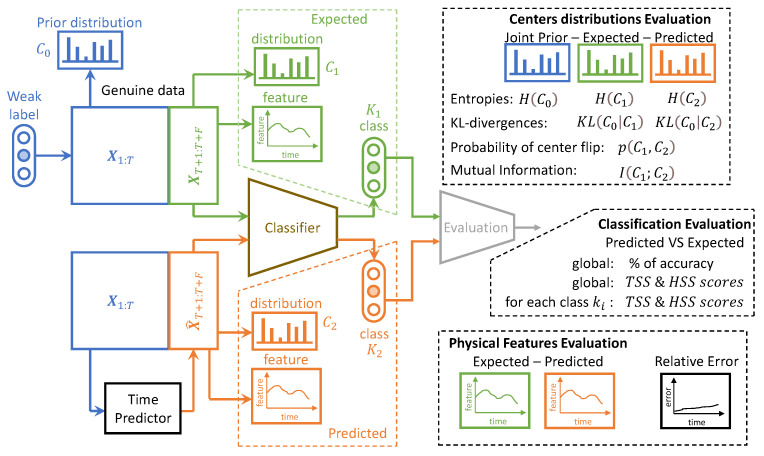
Evaluations performed on the proposed time predictor: center assignments, activity classification, and physical features. Classical MTS and CV evaluations were also performed without appearing in this diagram for readability concerns.

Considering that our work may be of interest to readers from various backgrounds, be it MTS prediction, information theory, ML in general, or applied physics, several types of evaluations performed on our model and concurrent ones are listed below; Figure 6 explains the last three listed evaluations.

- **MTS metrics:** MAE, MAPE, and RMSE evaluation. These metrics are defined at each time step as the means of $|X_{t}-{\hat{X}}_{t}|$ for MAE, $|X_{t}-{\hat{X}}_{t}\left|/\right|X_{t}|$ for MAPE, and the square root of the mean of ${\left(X_{t}-{\hat{X}}_{t}\right)}^{2}$ for RMSE.
- **CV metrics:** PSNR and SSIM evaluation. The ${\mathrm{PSNR}}_{t}$ is defined at each time step *t* as $-10\log_{10}\left({\mathrm{MSE}}_{t}\right)$, with ${\mathrm{MSE}}_{t}$ being the mean of ${\left(X_{t}-{\hat{X}}_{t}\right)}^{2}$. The larger the PSNR, the better the prediction. The SSIM is defined at each time step by [84]:
  (18) $$\begin{array}{c} SSIM_{t}=\frac{\left(2\mu_{t}\hat{\mu_{t}}+{\left(0.01L\right)}^{2}\right)\left(2\sigma_{t}\hat{\sigma_{t}}+{\left(0.03L\right)}^{2}\right)\left(cov_{t}+{\left(0.0212L\right)}^{2}\right)}{\left(\mu_{t}^{2}+{\hat{\mu_{t}}}^{2}+{\left(0.01L\right)}^{2}\right)\left(\sigma_{t}^{2}+{\hat{\sigma_{t}}}^{2}+{\left(0.03L\right)}^{2}\right)\left(\sigma_{t}\hat{\sigma_{t}}+{\left(0.0212L\right)}^{2}\right)}, \end{array}$$
  where *L* is the dynamic range of the pixel values, usually L=255; $\mu_{t}$ and $\sigma_{t}$ are the mean and standard deviations of all possible windows of length 7 in the time step data $X_{t}$, which are similar for ${\hat{\mu }}_{t}$ and ${\hat{\sigma }}_{t}$ for the predicted time step data ${\hat{X}}_{t}$. $cov_{t}$ is the covariance between all corresponding windows of length 7 on $X_{t}$ and ${\hat{X}}_{t}$. The SSIM has a maximum of 1 when $X_{t}={\hat{X}}_{t}$ and quantifies the visual structure present in the one-dimensional graph [84].
- **Astrophysical features** evaluation: Twelve features defined in [72] are evaluated for IRIS data. For these data, each time step corresponds to an observed spectral line in a particular region of the Sun. The intensity, triplet intensity, line center, line width, line asymmetry, total continuum, triplet emission, k/h ratio integrated, k/h ratio max, k-height, peak ratio, and peak separation are the twelve measures on these spectral lines. These features provide insight into the nature of physics occurring at the observed region of the Sun. These metrics are evaluated at each time to show that the IB principle and a powerful CV metric are sufficient to provide reliable predictions in terms of physics.
- **The IB evaluation** is performed on centroid distributions in the prior $X_{1:T}$, genuine $X_{T+1:T+F}$, and predicted forecasts ${\hat{X}}_{T+1:T+F}$. A *k*-means was performed in [55] for the spectral lines $X_{t}$ that are to be predicted over time. The corresponding centroids *C* are used in this work to evaluate information theory measurements on the quantized data. Entropies for the prior $H(c_{0})$, genuine $H(c_{1})$, and predicted $H(c_{2})$ distributions were averaged on the test data, and a comparison of the distributions between the prediction and the genuine was evaluated by computing the mutual information $I(c_{1};c_{2})$.
- The **classification accuracy** between the genuine and the forecast classifications was also evaluated. In the context of the IRIS data, three classes of solar activity are considered: QS, AR, and FL. Classifications are compared between the genuine target $X_{T+1:T+F}$ and predicted forecast ${\hat{X}}_{T+1:T+F}$, to assert whether the forecast activity complies with the targeted activity. TSS [76] and HSS [77] are evaluated globally and for each prediction class. These scores are defined in Section 2.8.

### 3.1. Evaluations of Predictions on IRIS Data

The model was trained on 240×240-sized images $X_{1:240}$ representing MTS with 240 time steps and 240 features at each time step, and the last 25% $X_{180:240}$ was masked at the input and predicted at the output. Each feature corresponds to a specific wavelength.

The data contain events of various durations. They were firstly partitioned into *training*, *validation,* and *testing* events; the model was tested on events that were not even partially seen at training time. All of the events were selected among those that last more than 240 time steps. Each event was divided into several 240×240 sized images $X_{1:240}$ and paired with the corresponding 240×240 masks.

The model was trained on 12,738 images of size 240×240; 25% of the right part of each image was used as the target for prediction. In the image, each spectrum (column) was normalized, such that the maximum value was 1; this is compliant with [55], where the 53 clusters were obtained after the normalization of the spectra. We directly applied the trained model in order to predict the time sequence with the half-duration continuation of the input; this is the *direct prediction* procedure. It was tested on 4490 *direct images*. To predict a longer continuation of the input, we adopted a straightforward common procedure and used a sliding 240×240 prediction window; this is the *iterated prediction* procedure. It was tested on 1962 *iterated images*. The evaluation was made by PSNR, SSIM, and the Hamming distance between the original and predicted corresponding cluster sequences. For this last metric, we adopted four different options. The cluster assignments are determined using a *k*-nearest-neighbor search at each time step, where the accuracy is measured by comparing if the data points have a common nearest centroid. NN${}_{1}$ refers to k=1 and NN${}_{4}$ refers to k=4.

**Figure 7:**
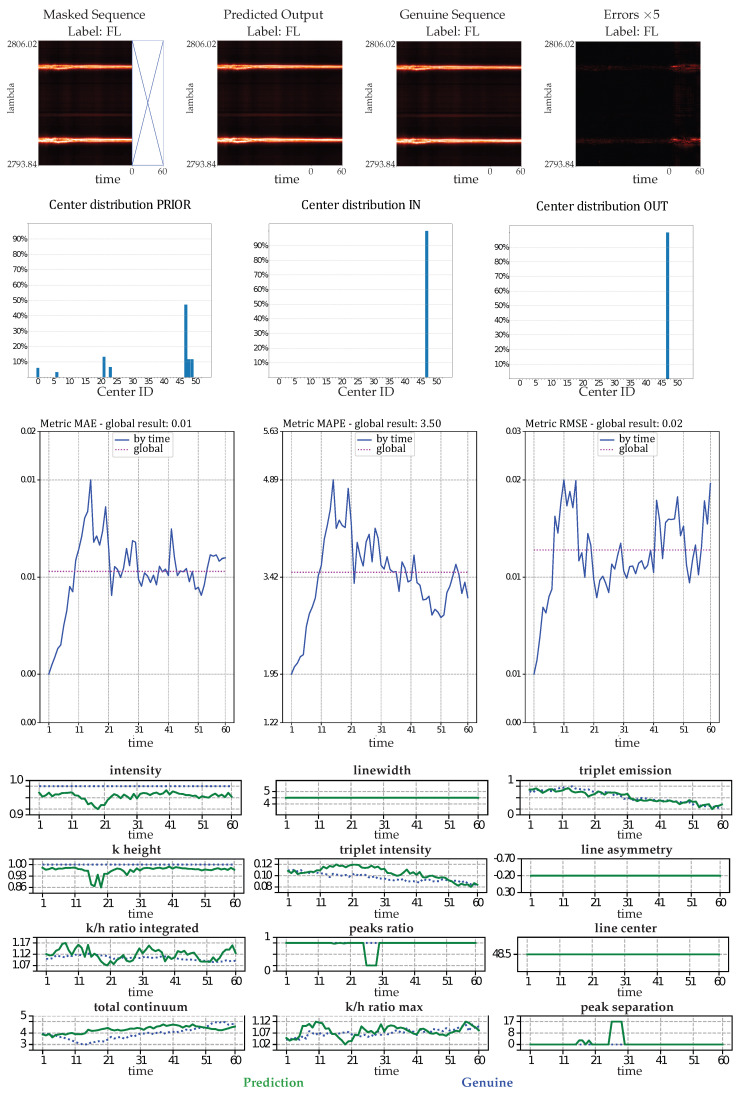
Evaluation of predictions for one flaring (FL) sample performed by the proposed IB-MTS model. The **first row** contains, respectively, the masked input, the predicted output, the genuine data, and the magnified pixel-wise error between the predicted and genuine. **Second row**: Spectral center distribution for the prior, the predicted, and the genuine MTS. **Third row**: MTS evaluation on the prediction. **Last twelve plots**: astrophysical features evaluations; the dotted blues represent the genuine and the green lines represent the prediction.

Figure 7 presents the forecast by the proposed IB-MTS model and its evaluations for one flaring (FL) sample. The first row of results shows that the genuine and predicted sequences look very similar, with a high PSNR of 35.65 dB. Although, some magnified differences can be naturally observed.

The second row shows results for the prior, genuine, and predicted sequences, in terms of the assignment at each time step to the NN centroid obtained from a k-mean procedure described in [55]. Although the prior distribution differs from the genuine sequence to be predicted, the model was able to generate a sequence that exhibited a similar physical pattern to the genuine one. This shows that the model seems to be able to predict astrophysical patterns even with a CV-based loss that does not specifically measure astrophysical features.

The third row of results evaluates the usual MTS metrics for this specific FL sequence prediction. The three metrics (MAE, MAPE, and RMSE) have small averages over the forecasted times. Interestingly, the MTS errors are very small and below the averages for the first ten predicted time steps. This could be attributed to the temporal proximity with the prior information, as well as the combination of convolutions with the $L_{tv}$ component of the loss defined in Equation (13), which softens the transition between the prior and the predicted data.

The last twelve plots show the time evolution of the astrophysical features extracted from the genuine (in blue) and predicted (in green) sequences. They show that the genuine features are predicted with more or less errors over time, but the predictions tend to follow the patterns of the genuine sequences. As these features were not considered in the loss used, this shows a non-negligible correlation between astrophysical features and CV metrics that were used to train the model.

#### Longer Predictions

For longer predictions, a standard well-known method performs recursive forecasts on data forecasted by the model. Under this setup, the prior MTS is a previously performed forecast, and one can legitimately expect an accumulation of errors. This is historically justified by the usage of one-step-forward models, such as the LSTM.

**Figure 8:**
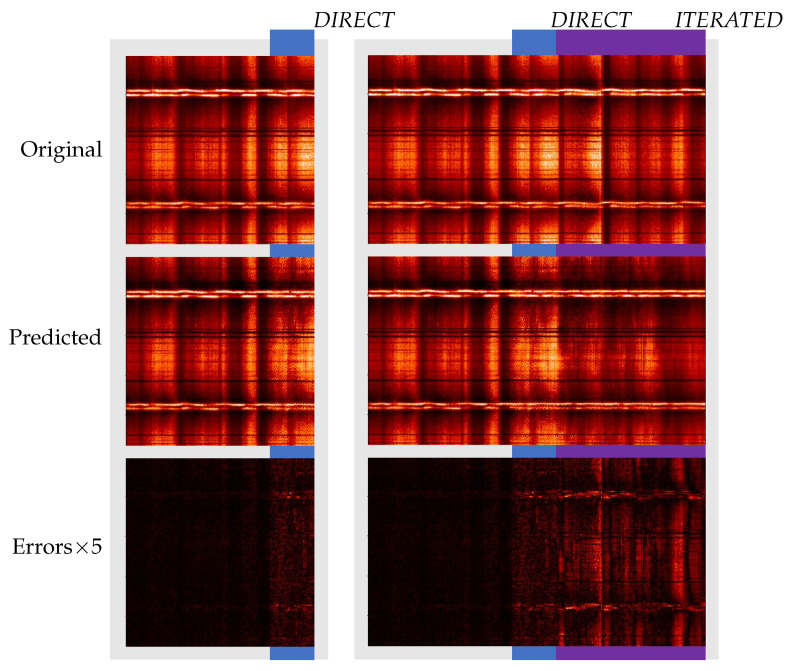
Prediction results: The first column presents the results of the direct predictions (blue part) and the second column presents the iterated predictions (violet part). A masked sample is given from the original sequence (**first row**); the prediction (**second row**) and the magnified (×5) differences (**third row**) are shown.

Figure 8 presents the short and long forecasts performed on a normalized IRIS quiet Sun (QS) sample. We can visually see that our approach is able to predict direct predictions as well as correct patters when we iterate the predictions on already predicted data.

### 3.2. MTS Metrics Evaluation

Table 2 presents the results of the evaluations of MAE, MAPE, and RMSE on IRIS, AR, and PB data. These metrics are averaged over all of the spectral and spatial dimensions, and all of the predicted time steps; for IRIS data, these metrics are also averaged over all types of solar activities. The model was trained and evaluated on each individual dataset. For all three metrics, our model outperforms the concurrent ones on the three datasets.

**Figure 9:**
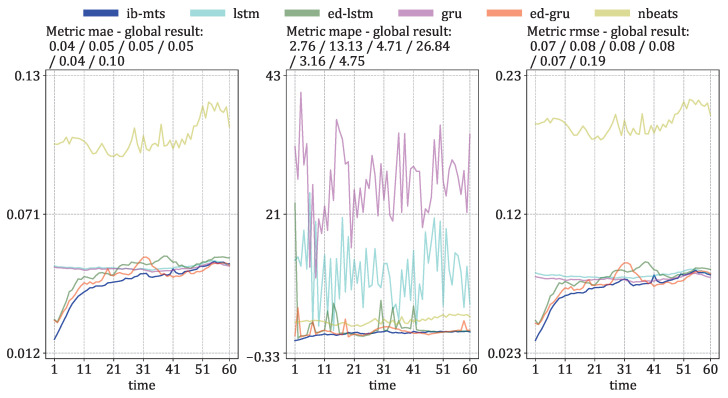
MTS metrics evaluation averaged on the test set for the direct prediction setups on QS, AR, and FL IRIS data.

**Figure 10:**
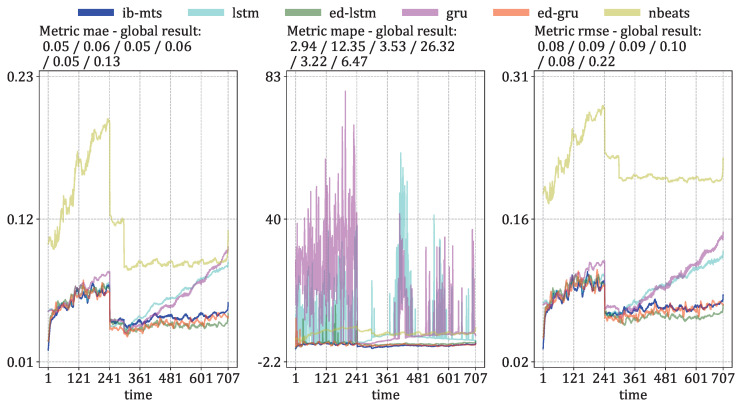
MTS metrics evaluation averaged on the test set for the iterated prediction setups on QS, AR, and FL IRIS data.

The descriptive temporal evolutions of these metrics are given in Figure 9 for the prediction errors under the *direct* setup, and in Figure 10 for the prediction errors under the *iterated* setup.

For the *direct* setup of Figure 9, our proposed model performs better than all of the concurrent ones in more than half of the time steps, and there is even a gap in the performance for the first 30 time steps. The traditional LSTM and GRU have very bad performances in the first 20 time steps compared to the other models. The three models with IB formulations, which propose compressions of the time dimension similar to ED-LSTM and ED-GRU, demonstrate similar performances with a performance gap in the first 20 time steps compared to the proposed IB-MTS. However, ED-GRU manages to recover this gap and even performs slightly better in the last 20 time steps in terms of MAE and RMSE. NBeats does not perform well on the IRIS dataset; the results for MAE and RMSE are among the worst, whereas the MAPE results are average. This means that NBeats produces predictions with significant errors for high values, but it is highly accurate for small values. One reason for this could be the small number of trainable parameters for NBeats, compared with the complexity of the IRIS dataset. In simpler datasets, such as AL and PB, NBeats can achieve the second-best results after IB-MTS and the best results on iterated predictions on the PB dataset. One possible explanation for the weak performances of NBeats on IRIS datasets could be the complexity and the strong spatiotemporal dependencies of spectral data, where the spatiotemporal dependencies are less clear on AL and PB data, with sensors being sorted by their latitudes, ignoring their longitudes. For the MAPE metric, the proposed IB-MTS still performs better than the other ones, even with the last time steps. One interesting fact is that even if the performances are quite far in the first part of the time steps, the classical LSTM and GRU seem to have fewer error variabilities than the ED-LSTM and ED-GRU for MAE and RMSE metrics. This could be explained by the specific designs of NBeats for long-term predictions, suggesting that other currently designed IB models may close the performance gap over the long term while still providing comparable results. In contrast to these conclusions, concerning the MAPE metric, the classical LSTM and GRU models have a lot of error variability and perform worse than the IB-designed models. The variability could be due to larger errors for small values because these types of errors have significant impacts on the MAPE metric.

**Figure A1:**
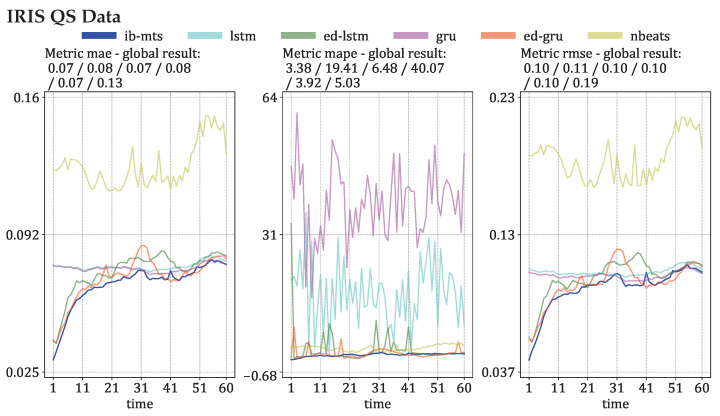

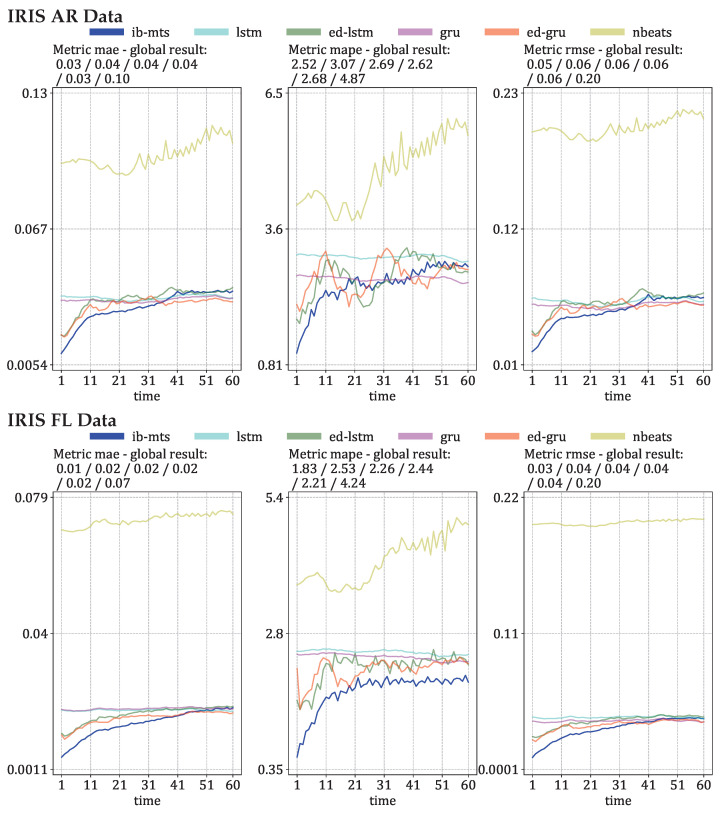
Detailed MTS metrics evaluation on the test set for the direct prediction setup. The evaluations are given for each solar activity: the first row of results is for QS activity, the second row is for AR, and the last row is for FL.

Concerning the last 20 time steps, Figure A1 from Appendix C can provide some explanation for the gain of performance in terms of the MAE and RMSE of the ED-GRU. This figure provides details of evaluations on the three different types of data included in the training and test data, i.e., QS, AR, and FL data, representing, respectively, 45.6%, 26.2%, and 28.2% of the (short) data. The figure shows that our proposed IB-MTS performs better than all of the others in all of the time steps for QS data, and is the most represented class in the trained and tested data. The other IB models still perform worse than IB-MTS for the first 30 time steps but they perform better in terms of MAE and RMSE in AR and FL data for the last 20 time steps.

Figure 10 presents the evaluations of the MTS metrics in the *iterated* setup, where the model predicts 60 time steps ahead on data that have already been predicted. For this procedure, the proposed IB-MTS does not show a specific improvement in performance, except for the MAPE metric and the first 20 time steps, where the first *direct* prediction is performed. It is also important to note that the traditional LSTM and GRU models have high accumulated errors over time and perform much worse than the others in the last 200 time steps. The specific drop in the curves at 241 time steps is directly related to the variation in the length of the MTS within the testing set.

**Figure 11:**
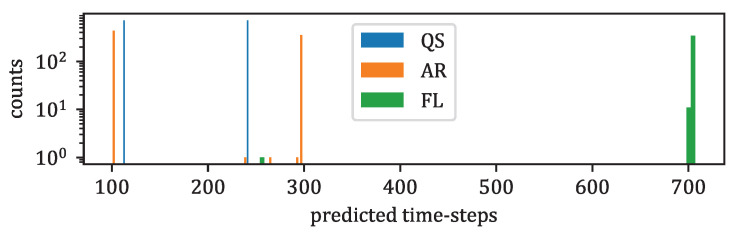
Histogram of the event durations from IRIS data.

Figure 11 shows a histogram of the number of QS, AR, and FL data present in the test data by the number of total time steps; the majority of FL data have predicted durations greater than 700 time steps. On the contrary, the majority of QS and AR data have predicted durations smaller than 300 time steps.

**Figure A2:**
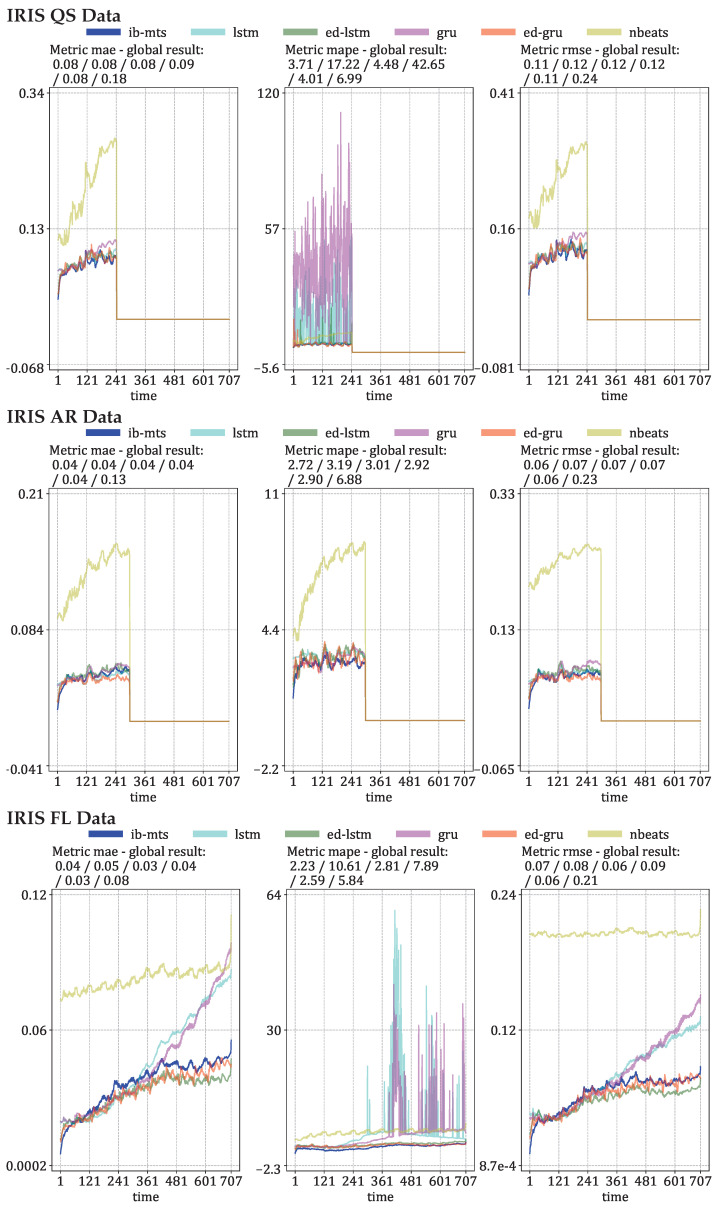
Detailed MTS metrics evaluation on the test set for the iterated prediction setup. The evaluations are given for each solar activity: the first row of results is for QS activity, the second row is for AR, and the last row is for FL.

Figure A2 from Appendix C presents a detailed comparison of the MTS evaluations for each class of *iterated* data. These graphs firstly explain the specific shapes present in Figure 10 by the fact that no QS data are present after 242 predicted time steps and no AR data are present after 298 predicted time steps. The proposed IB-MTS still performs very well on *iterated* QS data for all of the metrics, as well as for the MAPE metric on all types of data. ED-LSTM and ED-GRU perform better than IB-MTS after 120 time steps for MAE and RMSE metrics. One interpretation of this could be that the proposed IB-MTS is not designed to handle predictions on predictions, and does not include a state channel, such as in LSTM of GRU. Moreover, many variabilities are present for LSTM and GRU on the MAPE metric, whereas the IB-MTS model remains consistently stable and outperforms the other models in all scenarios. This could be attributed to the errors present for small values because they impact a lot of the results of the MAPE. In fact, an error of 0.1 for a value of 0.2 has a MAPE of 100×0.1/0.2=50, whereas the same error of 0.1 for a value of 0.8 has a MAPE of 100×0.1/0.8=12.5.

### 3.3. Computer Vision Metrics Evaluation

For the two procedures, *direct* and *iterated*, as described in Section 3.1, Table 3 provides the results of the evaluations in terms of PSNR and SSIM averaged on the test data. The proposed IB-MTS performs better than the concurrent models on IRIS data for both procedures. The gain of performance is very sensible for *direct* predictions. For *iterated* predictions, the results are more grouped and the ED-GRU performs similar to IB-MTS. On AL data, IB-MTS also outperforms concurrent models, except for the PSNR on *direct* data, where ED-LSTM performs a bit better.

NBeats still fails on IRIS data for the CV metric evaluation but performs well on the simpler datasets (AL and PB). NBeats can even achieve the highest SSIM on PB data. Yet our proposed IB-MTS model is the most stable in terms of the results on various datasets and it outperforms the concurrent ones on almost all metrics and datasets. NBeats also performs the best for the *iterated* procedure on PB data. Our proposed IB-MTS model was designed to predict multiple steps ahead in a *direct* fashion and does not generally extend very well for the *iterated* procedure.

**Figure 12:**
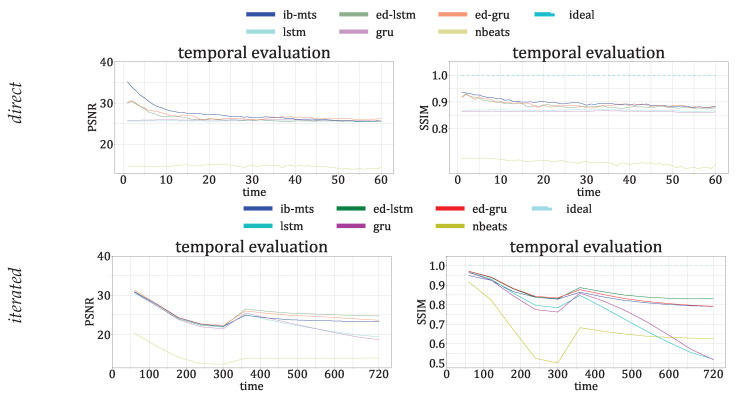
CV evaluation (over time) of the forecast for the direct and iterated predictions on IRIS data.

The first row of Figure 12 provides detailed time evolutions of these metrics on IRIS *direct* data. They show a sensible gain of performance for the first 20 time steps, whereas the classical LSTM and GRU models do not have specific time evolutions in terms of performance and they perform worse than the others.

The second row of Figure 12 provides detailed time evolutions of the PSNR and SSIM metrics on the IRIS *iterated* data. ED-LSTM and ED-GRU perform a bit better than IB-MTS under the *iterated* setup for more than 300 time steps whereas the classical LSTM and GRU models perform worse. The specific variation of the curve at 300 time steps is explained by the diversity of the MTS durations evoked in Figure 11.

#### 3.3.1. Information Bottleneck Evaluation on IRIS Data

This subsection evaluates IT-related measures on IRIS prior $X_{1:180}$, genuine $X_{181:240}$, and predicted ${\tilde{X}}_{181:240}$ data, as explained in Figure 6. Entropies H(·), KL-divergences KL(·||·), and mutual information I(·,·) are estimated by using the centroids obtained by a version of the k-means process performed in [55]. The dictionary of these 53 centroids is publicly available (i4ds.github.io/IRISreader/html/centroiddata.html, accessed on 20 February 2023). Each prior sequence $X_{1:180}$ contains 180 centroids, one for each time step, with repetitions because the dictionary of centroids only has 53 centroids. In the same manner, each genuine $X_{181:240}$ and predicted ${\tilde{X}}_{181:240}$ sequence contains 60 centroids, with repetitions.

**Figure 13:**
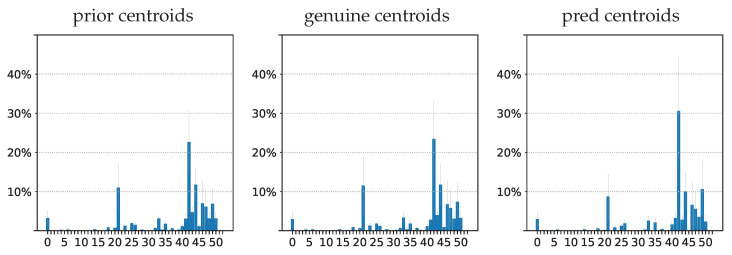
Average distributions of centroids with their standard deviations as vertical gray error bars. The first graph is for the average prior central data, the middle graph is for the average genuine target, and the right graph is the average distribution of predictions performed with IB-MTS.

**Figure A3:**
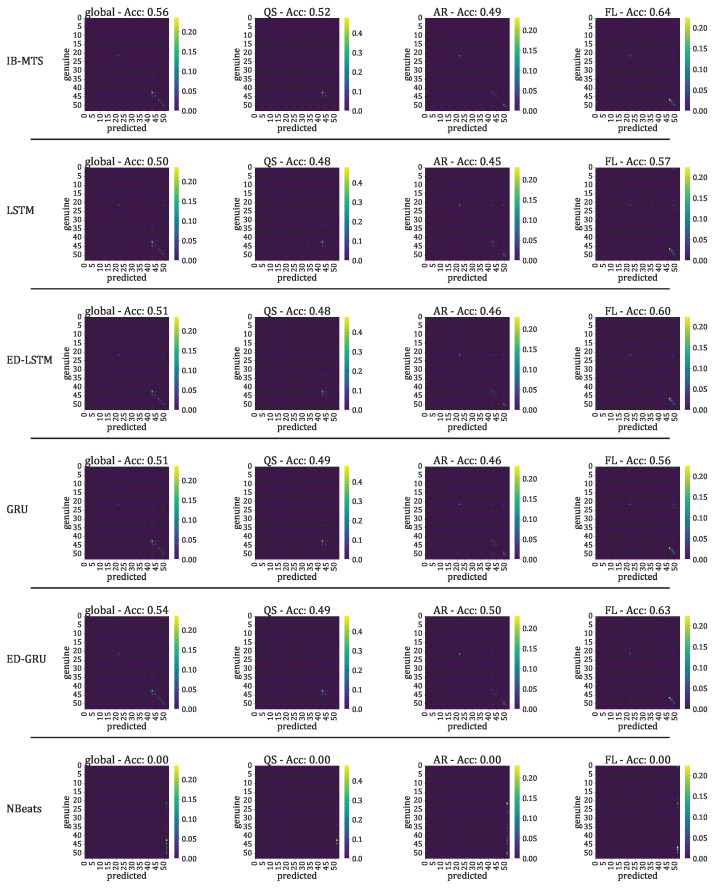
Confusion matrices for the prediction of centroids on IRIS data, for the direct procedure. We used the 53 centroids from [55]. Each row of results corresponds to a model. Columns are organized by data labels: *global* aggregate results for QS, AR, and FL data; other columns present the result for of each label, taken separately. Each confusion matrix gives results in terms of join probability distribution values between the genuine and the predicted. Probability values are displayed with color maps, where violet is the lowest probability and yellow is the highest.

**Figure A4:**
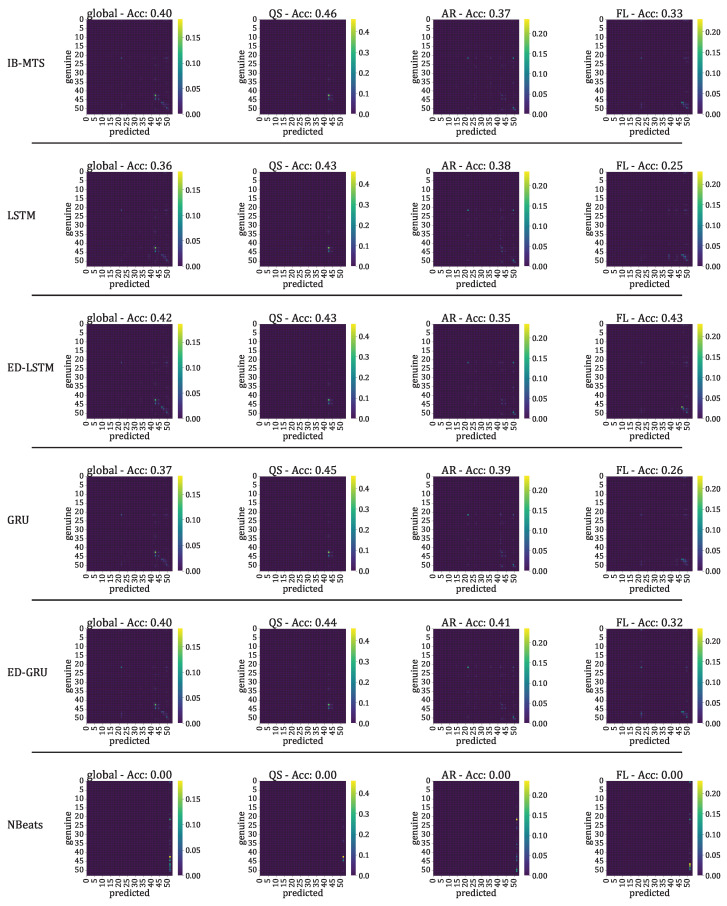
Confusion matrices for the prediction of centroids on IRIS data, for the iterated procedure. We used the 53 centroids from [55]. Each row of results corresponds to a model. Columns are organized by data labels: *global* aggregate results for QS, AR, and FL data; other columns present the results for of each label, taken separately. Each confusion matrix provides results in terms of join probability distribution values, between the genuine and the predicted. Probability values are displayed with color maps, where violet is the lowest probability and yellow is the highest.

Figure 13 shows the average distributions for prior, genuine, and predicted data. As expected, the average prior and genuine distributions of centroids are very similar and correspond to averages of the observed data. The average distribution of the predicted data remains close to that of the observed data, with only a few deviations in means and standard deviations. In particularly, centroid numbers 42 and 49 are a bit over-represented in the predictions whereas centroid numbers 20 and 44 are a bit under-represented. More details are given in Appendix C with the joint probabilities $p(c_{1},c_{2})$ of the centers present in the genuine $c_{1}$ and pred $c_{2}$ forecasts. Figure A3 presents the direct setup of IRIS data and Figure A4 presents the iterated setup. High probabilities on the diagonal $c_{1}=c_{2}$ indicate a high accuracy of the prediction in terms of centroids. These figures show that our proposed IB-MTS ensures the high conservation of the physics behind the spectra, whereas NBeats totally fails in this task.

Table 4 presents the average IT measurements estimated on the prior, genuine, and predicted data. $c_{0}$ stands for the centroids present in prior data, $c_{1}$ and $c_{2}$ are the ones present in genuine and predicted data. Ideally, $KL(c_{0}\left|\right|c_{2})=KL(c_{0}\left|\right|c_{1})$, and $H\left(c_{2}\right)=H\left(c_{1}\right)=I\left(c_{1},c_{2}\right)$. The higher the $I(c_{1};c_{2})$, the better. The results show that IB-MTS predicts the best $c_{2}$ centroids. This is confirmed by the highest value of mutual information $I(c_{1};c_{2})$ between the genuine and prediction data. Moreover, the KL-divergence $KL(c0||c_{2})$ between the prior and the prediction data is the closest to $KL(c0||c_{1})$ between the prior and the genuine data for IB-MTS on *direct* data. The interpretation of a high I(c1;c2) is that the IB-MTS model is the best at predicting the information present in the prediction, even without the need for a lot of extra information beyond what is given in the prior, because $KL(c_{0}||c_{2})$ is the lowest and closest to $KL(c_{0}||c_{1})$. As a consequence, it seems that IB-MTS is the model that best adheres to the IB principles. NBeats failed to predict the correct centroids. The KL-divergence $KL(c0||c_{2})$ between the prior and the prediction is the closest to $KL(c0||c_{1})$ but equal to 0; the mutual information $I(c_{1};c_{2})$ between the genuine and the prediction is 0, which means that, on average, it could not predict the information of the target. One reason for this could be that the intensity information is very important for the NBeats process on IRIS data. The fact that we removed this information from the dataset by normalizing each time step by the maximum values may penalize NBeats on the IRIS data, which is not the case with AL and PB data, where NBeats performs fine.

Concerning the *iterated* data, the highest values of mutual information $I(c1;c_{2})$ are still obtained by the IB models. IB-MTS can achieve the highest mutual information $I(c_{1},c_{2})$ with the lowest $KL(c_{0}||c_{1})$.

#### 3.3.2. Astrophysical Evaluations

A public dictionary of 53 centroids obtained by a version of k-means was performed on IRIS Mgh&k data [55]. Moreover, astrophysical features defined in [72] allow interpretability of Mghk spectra. This part evaluates the correspondence between centroid assignments for each genuine and predicted time step, and also evaluates the relative time evolution of the error between the genuine and predicted time steps. A k-NN search was performed between the genuine spectra at a given time step and the dictionary consisting of 53 centroids. The same search was performed for the same time step between the predicted spectra and the dictionary, and two sets of k centroids were compared. The considered time steps are successful *k*-NN assignments when they have at least one center in common.

For the two procedures (*direct* and *iterated*) described in Section 3.1, Table 5 provides the results of the prediction in terms of *k*-NN metrics averaged on the test data. For comparison, in the third column, we provide the accuracies of a theoretical worst model, which randomly assigns the *k*-nearest-neighbors among the 53 found in [55]. The most pessimistic accuracy for random classifications is obtained by the following combinatorial calculation:

(19) Randomk-NN=53k−53−kk53k.

**Figure 14:**
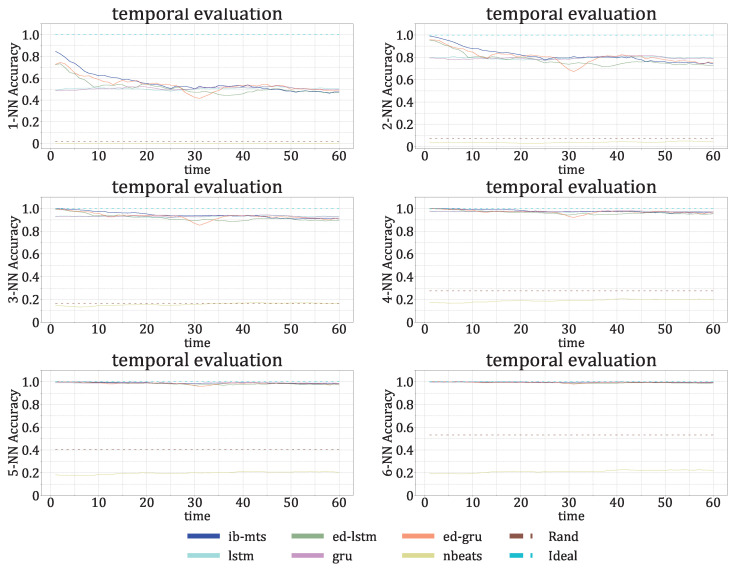
IRIS center assignment evaluation (over time) of the forecasts for direct predictions.

For k=2, the proposed IB-MTS model can already predict the sequence of clusters with 82% of accuracy, and even 99% for k=5, whereas a random assignment would give corresponding accuracies of 7.5% and 40%. The NBeats model fails on IRIS data, performing accuracies lower than the random assignments and not being able to predict spectra with the same centroid assignment as the genuine. Figure 14 presents the time evolution of the average k-NN accuracy for the *direct* procedure. There is a clear gain in performance for IB-MTS in the first 20 time steps for 1-NN and 2-NN accuracies. When *k* is greater than 4, the accounted performances are very similar for all time steps.

**Figure 15:**
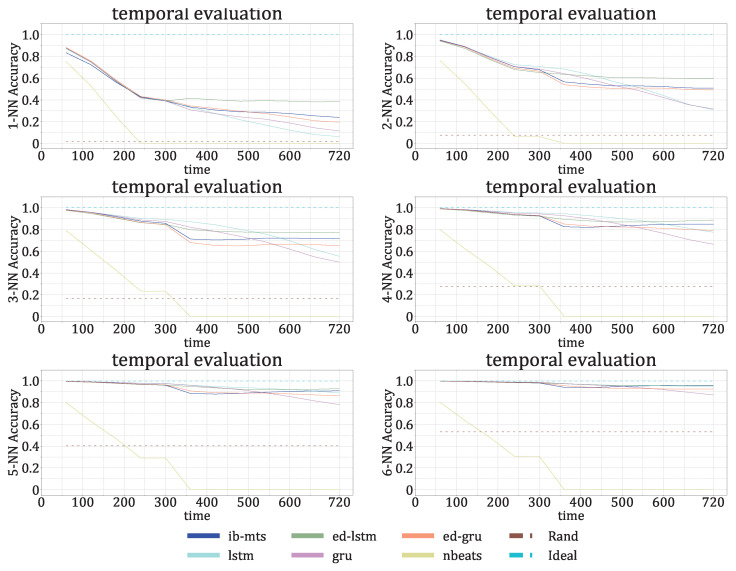
IRIS center assignment evaluation (over time) of the forecasts for iterated predictions.

Figure 15 presents the time evolution of the average k-NN accuracy for the *iterated* procedure. Similar to the other metrics, the IB-MTS model shows an average performance for the *iterated* procedure, while the other IB models and NBeats perform better when predictions are made iteratively on predicted data.

Table 6 provides detailed data on 1-NN center assignment accuracy, as well as HSS and TSS metrics, broken down by label and aggregated for all the data. Concerning the *direct* procedure, IB-MTS performs the best on each label, as well as overall, and ED-GRU obtains comparable results on AR data only. The results for the *iterated* procedure show that IB-MTS performs less favorably compared to other IB models.

**Figure 16:**
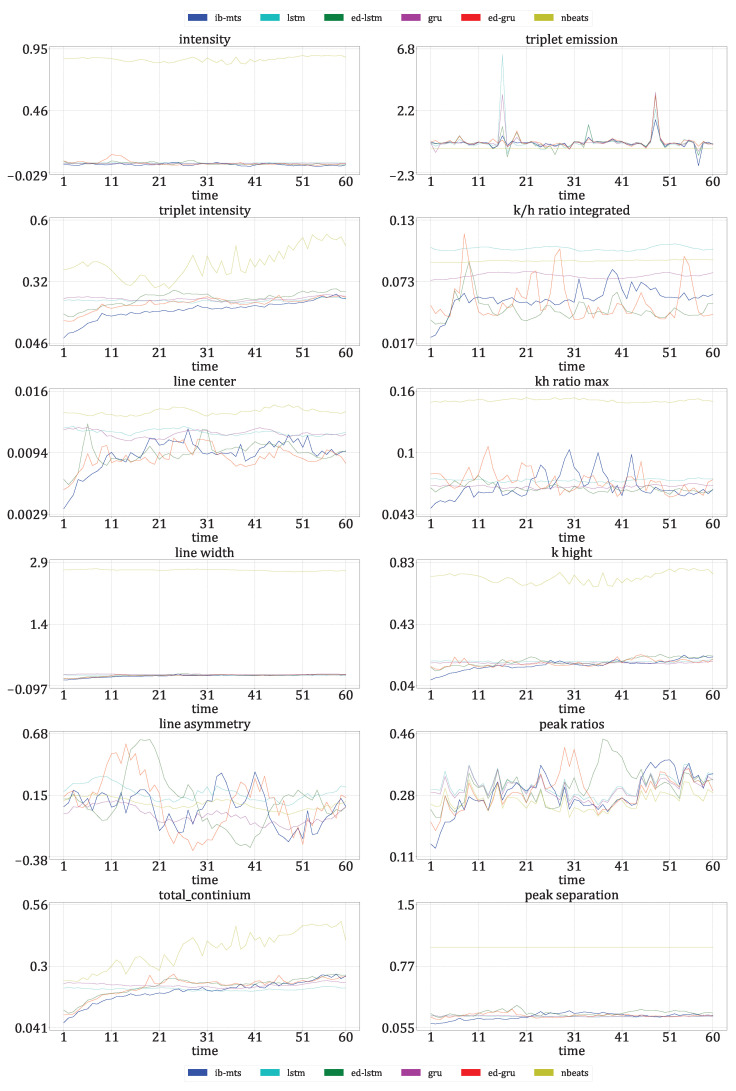
Evaluation of the relative prediction errors for physical features over time of the forecasts for IRIS data and the *direct* setup. The lower the better.

The time evolutions of the average relative errors for the astrophysical features are given in Figure 16. Given that each feature of a spectrum is a scalar output of a deterministic function $f_{k}\left(\cdot \right)$, these relative errors are defined at each time step by:

(20) $$ref_{k,t}=\frac{|f_{k}\left(X_{t}\right)-f_{k}\left({\tilde{X}}_{t}\right)|}{f_{k}\left(X_{t}\right)},$$

where ${\tilde{X}}_{t}$ is the time step *t* estimation of X by the model. IB-MTS is able to predict the physical features relatively well, with a significant gain of performance in the first 20 time steps in almost all time steps. This is an important result because features, such as the line center, k/h ratios, k-height, and peak separation are related to the positions of specific local maximums on the spectra, and the corresponding functions are not differentiable. We provide experimental proof that IB-MTS is able to predict features that are not easy to integrate in the loss of a deep model. NBeats have high errors in predicting intensities. This is coherent with the comments formulated in Section 3.3.1. It seems that NBeats cannot function without intensity information on IRIS data, which could be the reason for its failure on these data.

**Figure 17:**
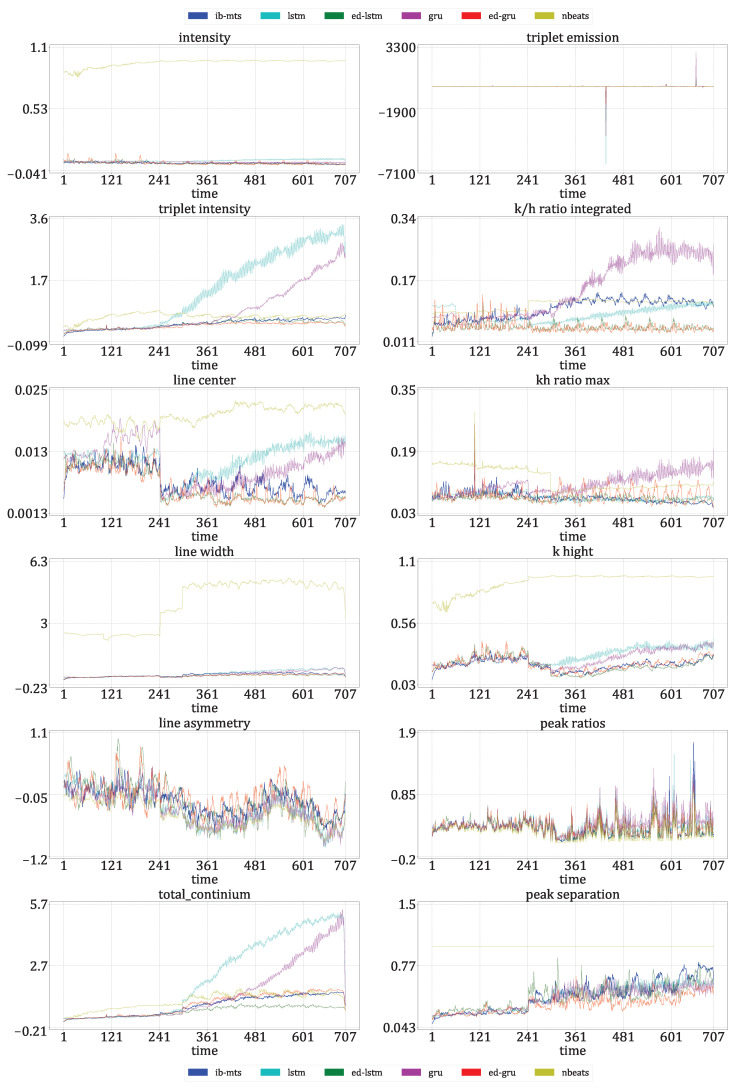
Evaluation of the relative prediction errors for physical features over time of the forecasts for IRIS data and the *iterated* setup.

Figure 17 shows the time estimations for the relative errors of features under the *iterated* setup. IB-MTS has an average performance under this setup, other IB models perform better, and classical LSTM and GRU performing worse.

#### 3.3.3. Solar Activity Classification

This section investigates the solar activity classifications of test data. Part of the IRIS Mghk data were labeled by the type of activity, such as QS, AR, and FL. The labeling was performed globally for each time sequence and not at each time step, such that if an event presents a flaring FL episode between time steps $t_{0}$ and $t_{1}$, the sample $X_{1:T+F}$ will be labeled as FL ($1<t_{0}<t_{1}<T+F$).

A previously trained classifier was used to classify the genuine and predicted sequences, $X_{T+1:T+F}$ and ${\tilde{X}}_{T+1:T+F}$. This classifier outputs a vector of size 3 with a categorical assignment. The assigned class corresponds to the minimum of the cosine pseudo-distance between the output of the classifier and the one-hot encoded version of the class. The classification is considered successful when they match. For the *iterated* procedure, the classification is estimated by the minimum cosine pseudo-distance with the average of the categorical outputs obtained by the classifier at each iteration. Table 7 shows the results of the classification in terms of percentage accuracy, HSS, and TSS metrics. For the short and long procedures, there are no specific performance gains for IB-MTS compared to the concurrent models. All models show high activity classification performances, except NBeats; previous results sections showed that the model failed to predict on the IRIS normalized data. IB-MTS has slightly higher TSS and HSS scores than other models.

## 4. Discussion

### 4.1. Conclusions

Our proposed model is not perfectly fine-tuned but it already shows very competitive results. Moreover, the integration of the total loss of $L_{1}$ and $L_{2}$ from Equation (6) will allow for better optimization of the full IB loss from Equation (4). The main goal of this paper was to provide a new theoretical formulation of the IB in the context of MTS, i.e., to bring theoretical and empirical proofs that are not only problems of multiple successive IBs, but unique, joint spatiotemporal IBs. As a consequence, these results and comparisons are convincing and support the presented IB formulation for MTS forecasting. Moreover, we show that IB models, which utilize compression through encoding and decoding of the time dimension, also perform better than classical recurrent models.

The gain of performance is also very significant for the first 20 time steps across almost all evaluated metrics. This may be due to the combination of convolutions and the transition term $L_{tv}$ present in the loss in Equation (13), which smooths out the predictions at the border for the first predicted time steps.

### 4.2. Spatial Sorting of MTS Data

Spatial dimensions in AL and PB are sorted by increasing latitudes, while the IRIS spatial dimension is sorted by increasing the wavelength. Other possible orderings for AL and PB datasets involve increasing longitudes or distances from a specific coordinate. The spatial relationship of the time series in the AL dataset is closely associated with the spatial correlation of the weather and sunshine levels. The spatial relation of the time series in the PB dataset is linked to the connection with the road networks around San Francisco.

Other works [1,17,18,60] highlight the limitations of the models that only consider spatial and temporal dependencies separately. Spatiotemporal dependencies are locally modeled with convolutions on spatiotemporal pseudo-images. Moreover, the spatiotemporal dimensions are compressed by applying successive convolutions with a stride of 2 in the first half of the U-Net architecture. Because of this spatiotemporal compression structure, each hidden layer of the encoding part of the U-Net creates a more global model of the spatiotemporal dependencies, such that, in the end, the bottleneck models the global spatiotemporal dependencies. We did not test the results of permuting the natural spatial ordering of the datasets as it was not part of our research questions. However, we believe that such permutation would likely penalize the training of all models except for the simple LSTM and GRU models.

The bottleneck of our proposed model has 1×1 spatiotemporal dimensions. This extreme shrinking of spatiotemporal dimensions is crucial in our model. Firstly, it allows one to always obtain unmasked data in the bottleneck and predict by decoding the posterior that is masked at the source. Secondly, as explained above, it allows for the global modeling of the spatiotemporal dependencies of MTS.

### 4.3. Non-Homogeneous Cadences of IRIS Data

The proposed model achieves an extreme reduction of the spatiotemporal dimensions to 1×1 and *C* channels. We believe that this extreme spatiotemporal reduction helps to tackle the problem of the non-homogeneous cadences of the temporal dimension presented in Figure 4 because it is compressed into a unique spatiotemporal dimension in the bottleneck.

The exact theoretical explanation as to why the model performs well with non-homogeneous data still remains open and is the subject of investigation. Synthetic periodic data with non-homogeneous frequencies may help test the robustness of all models. In our paper, the convincing results on IRIS data show that our proposed model is robust to non-homogeneous data. We did not extend the study of this question to other datasets as we could not find any other MTS dataset with non-homogeneous cadences of observations. Usually, physical observations are designed with a fixed cadence to keep the analysis simple. As a consequence, we do not believe that the elaboration of synthetic non-homogeneous data is a priority for this work and we just showed the good performance of the proposed model on IRIS data.

In addition, the non-homogeneous cadences of observations in the IRIS data may contribute to the poor forecasting performance of NBeats for this dataset. NBeats is based on the decomposition of MTS data into interpretable MTS signals that compose the observations, similar to the trend and seasonality decomposition. It is very challenging and perhaps impossible to find the trend and seasonality of MTS observations with non-homogeneous cadences. In AL and PB data, the cadences were the same for all of the data, and NBeats performs much better; however, the best results are given by our proposed model for these data.

### 4.4. Pros

Interestingly, while the model is trained on a CV loss that is not designed to measure accuracies in activity classifications or astrophysical information, the model is still able to predict the information with significant accuracy. This is in favor of interpretability and we believe that this is a great plus.

Unlike most recent studies in MTS forecasting, where spatiotemporal embeddings are designed prior to being fed to the models, our model jointly works straight on spatiotemporal dimensions seen together as images. Because of the fully masked convolutional structure, we believe that such simplicity is the advantage of our model, which can be easily applied to data, and generalized to more dimensions, such as for videos where the spatial information is composed of two joint dimensions.

### 4.5. Cons and Possible Extensions

Because of the activations used, the models were tested on normalized data, where each time step had a maximum of one. While this is suitable for many applications where only the spatial distribution of the data is important at each time step, such as the shape of a spectral line, in some applications, it is also important to know the real maximum value or *intensity* of the data at each time step. We believe that in this case, intensity forecasting is a simpler issue than the one solved in our work; one can train a classical RNN model to predict the intensities in parallel to the spatiotemporal MTS.

Our model has much more parameters to train than classical RNNs, but still less than the most recent transformers. Without significant difficulties, we could train our model on data with 240×240 dimensions. Moreover, the performant CV loss used to train the model comes at the expense of memory comes at the expense of to save the parameters of the VGG-16 used in the loss.

We believe that the model could be further improved by integrating more interpretability and prediction of the forecast errors. These features were not targeted in this step and are not in the scope of this work.

### 4.6. Future Research Directions

Recent understandings and implementations of the IB principle could also further improve the performance of our presented IB-MTS. For instance, [39] showed that the general IB principle is equivalent to several adversarial and CV losses present at the output and at the compressed bottleneck levels, where we only considered the particular case with the CV loss at the output. In particular, introducing a loss at the bottleneck should help the compression and enhance data representations at the bottleneck, leading to increased interpretability. This approach would certainly bring new improvements, following the ones achieved by this work, showing the importance of the IB formulation in the context of MTS forecasting. Moreover, for a better estimation of the forecasting error, one could attempt to amend the output of the network and predict the means and standard deviations of Gaussian distributions for each predicted pixel instead of a singular value. This would make the model stochastic and facilitate the confidence estimation in the forecasted outputs.

## Figures and Tables

**Table 1:** Parameters and trained steps of the evaluated models.

	IB-MTS	LSTM	ED-LSTM	GRU	ED-GRU	N-BEATS
Parameters	65,714,097	461,760	401,840	347,040	308,640	100,800
Trained step (ms/sample)	91	83	98	87	98	424

**Table 2:** MTS metric results.

Data		Model	IB-MTS	LSTM	ED-LSTM	GRU	ED-GRU	NBeats
IRIS	*direct*	*MAE*	0.04	0.05	0.05	0.05	0.04	0.10
		*MAPE*	2.76	13.13	4.71	26.84	3.16	4.75
		*RMSE*	0.07	0.08	0.08	0.08	0.07	0.19
	*iterated*	*MAE*	0.05	0.06	0.05	0.06	0.05	0.13
		*MAPE*	2.94	12.35	3.53	26.32	3.22	6.47
		*RMSE*	0.08	0.09	0.09	0.10	0.08	0.22
AL	*direct*	*MAE*	0.08	0.10	0.08	0.10	0.09	0.11
		*MAPE*	3.71	5.27	4.58	5.50	5.20	6.56
		*RMSE*	0.15	0.16	0.14	0.16	0.16	0.18
	*iterated*	*MAE*	0.08	0.19	0.16	0.23	0.16	0.11
		*MAPE*	4.00	11.37	9.10	12.94	9.28	6.23
		*RMSE*	0.15	0.26	0.23	0.30	0.23	0.18
PB	*direct*	*MAE*	0.19	0.46	0.46	0.50	0.46	0.22
		*MAPE*	4.47	10.15	10.03	10.76	10.14	5.19
		*RMSE*	0.24	0.54	0.51	0.60	0.52	0.28
	*iterated*	*MAE*	0.24	0.45	0.45	0.45	0.45	0.23
		*MAPE*	4.23	10.00	9.98	10.01	9.98	5.51
		*RMSE*	0.30	0.51	0.500	0.51	0.50	0.28

**Table 3:** Accuracy in terms of average PSNR and SSIM for *direct* and *iterated* predictions.

Dataset		Metric	*IB-MTS*	*LSTM*	*ED-LSTM*	*GRU*	*ED-GRU*	*NBeats*
IRIS	*direct*	PSNR	**27.2**	25.6	26.3	25.9	26.7	14.6
		SSIM	0.897	0.869	0.887	0.864	0.891	0.673
	*iterated*	PSNR	23.8	23.0	23.4	22.8	23.8	13.4
		SSIM	0.868	0.821	0.864	0.809	0.868	0.586
AL	*direct*	PSNR	17.2	16.0	17.4	15.9	16.4	15.7
		SSIM	0.518	0.400	0.488	0.377	0.401	0.346
	*iterated*	PSNR	16.7	11.8	13.0	10.6	13.1	15.2
		SSIM	0.516	0.046	0.198	0.023	0.166	0.361
PB	*direct*	PSNR	12.5	5.4	6.0	4.5	5.7	11.4
		SSIM	0.361	0.013	0.000	0.004	0.000	0.470
	*iterated*	PSNR	10.5	5.9	6.0	5.9	6.0	11.0
		SSIM	0.235	0.003	0.003	0.003	0.004	0.472

**Table 4:** Information comparison between the prior centroids *c*0, the genuine centroids *c*1, and the predicted centroids *c*2 for the IRIS dataset. The results on *H*, KL, and *I* are averaged over all testing samples. $H(c_{0})$, $KL(c_{0}||c_{1})$, and $H(c_{1})$, being statistics on the prior and the genuine, do not depend on the method.

Dataset		Metric	*IB-MTS*	*LSTM*	*ED-LSTM*	*GRU*	*ED-GRU*	*NBeats*
IRIS	*direct*	$H(c_{0})$	3.922	3.922	3.922	3.922	3.922	3.922
		$KL(c_{0}||c_{1})$	0.005	0.005	0.005	0.005	0.005	0.005
		$KL(c_{0}||c_{2})$	0.111	0.311	0.111	0.344	0.198	0.000
		$H(c_{1})$	3.890	3.890	3.890	3.890	3.890	3.890
		$H(c_{2})$	3.567	3.382	3.579	3.280	3.465	0.000
		$I(c_{1};c_{2})$	1.753	1.607	1.613	1.597	1.681	0.000
	*iterated*	$H(c_{0})$	3.968	3.968	3.968	3.968	3.968	3.968
		$KL(c_{0}||c_{1})$	0.003	0.003	0.003	0.003	0.003	0.003
		$KL(c_{0}||c_{2})$	0.288	0.575	0.308	0.416	0.486	0.000
		$H(c_{1})$	3.957	3.957	3.957	3.957	3.957	3.957
		$H(c_{2})$	3.487	3.325	3.385	3.301	3.229	0.000
		$I(c_{1};c_{2})$	1.352	1.276	1.319	1.219	1.314	0.000

**Table 5:** Evaluation on IRIS data: Percentage accuracies in terms of *k*-NN for *direct* prediction of the sizes of the training data and *iterated* prediction using a basic sliding window approach. The random *k*-NN cluster assignment accuracy is given for comparison and corresponds to the worst that can be expected for each *k*-NN assignment.

	Metric	Rand${}_{k-\mathrm{NN}}$	IB-MTS	LSTM	ED-LSTM	GRU	ED-GRU	NBeats
*direct*	1-NN	1.9	55.7	50.5	51.4	50.8	54.1	0.0
	2-NN	7.5	81.9	79.5	77.8	79.4	80.5	3.8
	3-NN	16.3	94.3	93.0	91.8	93.2	93.1	15.8
	4-NN	27.6	97.7	97.3	96.3	97.4	97.0	19.0
	5-NN	40.3	98.9	98.8	98.2	98.9	98.6	19.7
	6-NN	53.2	99.6	99.5	99.1	99.5	99.4	21.1
*iterated*	1-NN	1.9	45.8	42.6	43.6	43.4	45.0	0.0
	2-NN	7.5	73.8	72.1	70.1	72.0	72.4	3.9
	3-NN	16.3	89.4	88.9	87.0	88.3	87.8	16.1
	4-NN	27.6	95.1	95.2	93.7	94.5	94.4	19.4
	5-NN	40.3	97.6	97.8	96.8	97.3	97.2	20.2
	6-NN	53.2	98.9	99.0	98.5	98.6	98.7	21.8

**Table 6:** Evaluation of 1-NN centroid assignment accuracy for the *direct* and *iterated* predictions.

Model	Metric	IB-MTS	LSTM	ED-LSTM	GRU	ED-GRU	N-BEATS
*direct*							
Global	% Accuracy	55.7	50.5	51.4	50.8	54.1	0.0
	TSS	0.49	0.43	0.45	0.43	0.47	0.00
	HSS	0.50	0.44	0.45	0.44	0.48	0.00
QS	% Accuracy	52.5	47.6	47.7	48.6	49.0	0.0
	TSS	0.26	0.18	0.21	0.19	0.22	0.00
	HSS	0.28	0.20	0.22	0.20	0.22	0.00
AR	% Accuracy	49.5	44.8	45.7	46.0	49.9	0.0
	TSS	0.43	0.37	0.39	0.39	0.43	0.00
	HSS	0.43	0.37	0.39	0.39	0.43	0.00
FL	% Accuracy	63.7	57.3	60.3	56.3	63.5	0.0
	TSS	0.58	0.51	0.53	0.49	0.57	0.00
	HSS	0.58	0.51	0.53	0.49	0.57	0.00
*iterated*							
Global	% Accuracy	40.4	36.4	41.8	37.4	40.0	0.0
	TSS	0.33	0.29	0.35	0.30	0.32	0.00
	HSS	0.34	0.29	0.35	0.30	0.32	0.00
QS	% Accuracy	46.3	43.4	42.7	44.7	43.9	0.0
	TSS	0.15	0.10	0.12	0.10	0.12	0.00
	HSS	0.17	0.11	0.13	0.12	0.14	0.00
AR	% Accuracy	37.2	38.2	34.8	39.0	41.3	0.0
	TSS	0.30	0.30	0.26	0.31	0.33	0.00
	HSS	0.30	0.30	0.26	0.31	0.33	0.00
FL	% Accuracy	33.0	24.8	42.6	26.3	31.8	0.0
	TSS	0.24	0.18	0.33	0.18	0.22	0.00
	HSS	0.24	0.17	0.33	0.17	0.22	0.00

**Table 7:** Accuracy of solar activity classifications for the predicted versus genuine MTS with the *direct* and *iterated* prediction setups.

Model (Count)	Metric	IB-MTS	LSTM	ED-LSTM	GRU	ED-GRU	N-BEATS
*direct*							
Global (8000)	% Acc	95	95	95	95	95	88
	TSS	0.911	0.911	0.906	0.910	0.909	0.805
	HSS	0.915	0.906	0.911	0.905	0.914	0.785
QS (3680)	% Acc	97	96	96	96	96	94
	TSS	0.938	0.911	0.916	0.910	0.918	0.876
	HSS	0.936	0.911	0.915	0.910	0.917	0.874
AR (536)	% Acc	96	96	97	96	97	91
	TSS	0.613	0.401	0.327	0.400	0.311	0.000
	HSS	0.640	0.371	0.366	0.362	0.349	0.000
FL (3784)	% Acc	98	99	98	99	99	92
	TSS	0.958	0.971	0.965	0.972	0.972	0.838
	HSS	0.959	0.971	0.965	0.972	0.972	0.843
*iterated*							
Global (8000)	% Acc	94	94	93	94	95	86
	TSS	0.979	0.899	0.870	0.896	0.895	0.768
	HSS	0.889	0.891	0.869	0.889	0.901	0.738
QS (3680)	% Acc	96	95	95	95	95	88
	TSS	0.914	0.903	0.891	0.892	0.907	0.777
	HSS	0.915	0.903	0.893	0.891	0.908	0.767
AR (536)	% Acc	94	95	95	96	96	92
	TSS	0.544	0.387	0.188	0.399	0.277	0.168
	HSS	0.594	0.331	0.185	0.346	0.311	0.113
FL (3784)	% Acc	98	98	97	98	98	91
	TSS	0.948	0.955	0.930	0.960	0.957	0.932
	HSS	0.949	0.957	0.930	0.962	0.957	0.920

## Data Availability

Publicly available datasets were analyzed in this study. The code used in this work and selected classified IRIS data can be found at github.com/DenisUllmann/IB-MTS, accessed on 20 February 2023. [IRIS] Author: NASA; IRIS is a NASA small explorer mission developed and operated by LMSA. LMission operations are conducted at the NASA Ames Research Center, and significant contributions to downlink communications are funded by the ESA and the Norwegian Space Centre. For more information, please visit iris.lmsal.com/search/, accessed on 20 February 2023. [AL] Author: The National Renewable Energy Laboratory, U.S. Department of Energy, Office of Energy Efficiency and Renewable Energy, operated by the Alliance for Sustainable Energy LLC; Solar Power Data for the year 2006 in Alabama at www.nrel.gov/grid/solar-power-data.html, accessed on 20 February 2023. [PB] Author: California State Transportation Agency (CalSTA) Performance Measurement System (PeMS) and Yaguang Li; PeMS-BAY traffic data [74] at dx.doi.org/10.5281/zenodo.5146275, accessed on 20 February 2023.

## Abbreviations

The following abbreviations are used in this manuscript:

AL	solar power dataset for the year 2006 in Alabama
AR	solar active region
ARIMA	autoregressive integrated moving average model
CV	computer vision
DNN	deep neural network
FL	solar flare
GNN	graph neural network
GRU	gated recurrent unit
HSS	Heidke skill score
IB	information bottleneck
IRIS	NASA’s interface region imaging spectrograph satellite
IT	information theory
LSTM	long short-term memory model
MAE	mean absolute error
MAPE	mean absolute percentage error
ML	machine learning
MOS	mean opinion score
MSE	mean square error
MTS	multiple time series
NLP	natural language processing
NN	neural network
PB	PeMS-BAY dataset
PC	partial convolution
PSNR	peak signal-to-noise ratio
QS	quiet Sun
RAM	random access memory
RGB	red–green–blue
RMSE	root mean square error
RNN	recurrent neural network
SSIM	structural similarity
TS	time series
TSS	true skill statistic

## Appendix A. Theory

**Table A1:** Summary table of the notations.

Random Variables	
X	generic spatiotemporal data
$\tilde{X}$	estimation of X
*T*	scalar duration of the prior sequence
*F*	scalar duration of the posterior sequence
*M*	spatial size of spatiotemporal data
$X_{t}$	multidimensional data at time step *t*
$X_{t}^{m}$	scalar value at time step *t* and spatial index *m*
$X_{1:T}=X_{1:T}^{1:M}$	prior sequence
${\tilde{X}}_{1:T}$	prior sequence estimation
$X_{T+1:T+F}=X_{T+1:T+F}^{1:M}$	posterior genuine sequence
${\tilde{X}}_{T+1:T+F}$	posterior genuine sequence estimation
$X_{1:T+F}=X_{1:T+F}^{1:M}$	full sequence
${\tilde{X}}_{1:T+F}$	full sequence estimation
Z	bottleneck
1:T→T+1:T+F	transition from prior to posterior
$Z_{ib\_tr}$	IB bottleneck for transition 1:T→T+1:T+F learning
$Z_{ib\_ae}$	IB bottleneck of AE
M	generic mask for spatiotemporal data
$M_{1:T}$	prior mask
$M_{T+1:T+F}$	posterior mask
$M_{1:T+F}$	full mask
$M_{ib\_tr}$	mask at the IB bottleneck for transition
	1:T→T+1:T+F learning
1 & $1_{len}$ & $1_{row\times col}$	vectors and matrices of ones, eventually
	with specified length len or row and col sizes.
0 & $0_{len}$ & $0_{row\times col}$	vectors and matrices of zeros, eventually
	with specified length len or row and col sizes.
*K* & K	scalar & categorical labels
$c_{0}$, $c_{1}$ & $c_{2}$	prior, genuine, and predicted centroid assignments
**Information Theory**	
$p_{D}$	data distribution
$p_{\Theta }$ & $p_{\Phi }$	(encoding/decoding) distribution with parameter (Θ/Φ)
$E_{p_{D}}\left[\cdot \right]$	mean by sampling from $p_{D}$
$E_{p_{\Theta }}\left[\cdot \right]$	mean by sampling from $p_{\Theta }\left(Z|X\right)$
H(·) & H(·,·)	generic entropy and cross-entropy
$H_{p_{\Theta }}$	entropy parametrized by the encoder
$H_{p_{\Theta },p_{\Phi }}$	cross-entropy parametrized by the encoder and the decoder
KL(·||·) & I(·;·)	KL-divergence & mutual information
$I_{\Theta }$ & $I_{\Phi }$	Encoding and decoding mutual information
**Layers & mappers**	
Id	Identity mapper
Concat	Concatenation of tensors
(−/B/P)Conv	(-/Binary/Partial) Convolutional layer
(−/B/P)DConv	(-/Binary/Partial) Deconvolutional layer
**Losses & metrics**	
L	generic loss
$\tilde{L}=L_{1}+L_{2}+L_{3}$	upper bound on the loss L
$L_{3}^{Lap}$	$L_{3}$ with Laplacian assumption of $p_{\Phi }\left(X_{T+1:T+F}|Z_{ib\_tr}\right)$
$L_{3}^{Lap,UNet}$	$L_{3}^{Lap}$ for a U-Net architecture
$ref_{k,t}$	relative error for feature *k* at time step *t*

**Proof** **of** **Equation** **(5).** This proof is highly inspired by [39] but without variational approximation considerations. Let us consider the loss defined in Equation (4) and let us simplify the TS notations that are not relevant to this proof:

(A1) $$\begin{array}{cc} L(\Theta ,\Phi ) & =I_{\Theta }\left(X_{1:T};Z_{ib\_tr}\right)-\beta I_{\Phi }\left(Z_{ib\_tr};X_{T+1:T+F}\right)=I_{\Theta }\left(X;Z\right)-\beta I_{\Phi }\left(Z;Y\right). \end{array}$$

The first term is mutual information that is parameterized by Θ, which represents the parameters of the encoding part. This term can be decomposed:

(A2) $$\begin{array}{cc} I_{\Theta }\left(X;Z\right) & =E_{p_{\Theta }\left(x,z\right)}\left[\log\frac{p_{\Theta }\left(z|x\right)p_{D}\left(x\right)}{p_{\Theta }\left(z\right)p_{D}\left(x\right)}\right]\\ \; & =E_{p_{\Theta }\left(z\right)}\left[\log p_{\Theta }\left(z\right)\right]-E_{p_{\Theta }\left(x,z\right)}\left[\log p_{\Theta }\left(z|x\right)\right]\\ \; & =H_{p_{\Theta }}\left(Z\right)-H_{p_{\Theta }}\left(Z|X\right). \end{array}$$

The second term of (A1) is the mutual information parametrized by Φ, which represents the parameters of decoding, and can be decomposed:

(A3) $$\begin{array}{cc} I_{\Phi }\left(Z;Y\right) & =E_{p_{\Phi }\left(z,y\right)}\left[\log\frac{p_{D}\left(y|z\right)p_{\Phi }\left(z\right)}{p_{D}\left(y\right)p_{\Phi }\left(z\right)}\right]\\ \; & =E_{p_{D}\left(y\right)}\left[\log p_{D}\left(y\right)\right]-E_{p_{\Phi }\left(z,y\right)}\left[\log p_{D}\left(y|z\right)\right]\\ \; & =H_{p_{D}}\left(Y\right)-E_{p_{\Phi }\left(z,y\right)}\left[\log p_{D}\left(y|z\right)\frac{p_{\Phi }\left(y|z\right)}{p_{\Phi }\left(y|z\right)}\right]\\ \; & =H_{p_{D}}\left(Y\right)-E_{p_{D}\left(x,y\right)}\left[E_{p_{\Theta }\left(z|x\right)}\left[\log p_{\Phi }\left(y|z\right)\right]\right]+E_{p_{\Phi }\left(z,y\right)}\left[\log\frac{p_{\Phi }\left(y|z\right)}{p_{D}\left(y|z\right)}\right]\\ \; & =H_{p_{D}}\left(Y\right)-H_{p_{\Theta ,\Phi }}\left(Y|Z\right)+KL\left(p_{\Phi }\left(y|z\right)\left|\right|p_{D}\left(y|z\right)\right)\\ \; & \geq H_{p_{D}}\left(Y\right)-H_{p_{\Theta ,\Phi }}\left(Y|Z\right), \end{array}$$

because the Kullback–Leibler divergence $KL\left(p_{\Phi }\left(y|z\right)\left|\right|p_{D}\left(y|z\right)\right)$ is positive. As a consequence, by putting the results of Equations (A2) and (A3) in Equation (A1), one can obtain the following upper bound on the loss:

(A4) $$L\left(\Theta ,\Phi \right)\leq H_{p_{\Theta }}\left(Z\right)-H_{p_{\Theta }}\left(Z|X\right)+\beta H_{p_{\Theta ,\Phi }}\left(Y|Z\right)-\beta H_{p_{D}}\left(Y\right),$$

and $H_{p_{D}}\left(Y\right)$ being fixed by the data, the upper bound on the loss can be reduced to:

(A5) $$\tilde{L}\left(\Theta ,\Phi \right)=H_{p_{\Theta }}\left(Z\right)-H_{p_{\Theta }}\left(Z|X\right)+\beta H_{p_{\Theta ,\Phi }}\left(Y|Z\right),$$

□

**Remark** **A1** **(Details on** PConv **and** PDeconv **for MTS).** 
*With T representing the time steps of prior values and F representing the time steps of forecasted values, let us recall Equation (4):*

(A6)
$$L\left(\Theta ,\Phi \right)=I_{\Theta }\left(X_{1:T};Z_{ib\_tr}\right)-\beta I_{\Phi }\left(Z_{ib\_tr};X_{T+1:T+F}\right).$$

*This corresponds to the general formulation of the IB principle with the MTS notation. The bottleneck variable $Z_{ib\_tr}$ should then hold part of the information of prior time steps 1:T and forecast time steps T+1:T+F in order to learn the time transition. Equation (A6) can be rewritten using temporal masks on the source $X_{1:T+F}=Concat\left[X_{1:T},X_{T+1:T+F}\right]$:*

(A7)
$$\begin{array}{cc} L(\Theta ,\Phi )= & \underset{A}{\underset{︸}{I_{\Theta }\left(\left[X_{1:T},X_{T+1:T+F}\right]⊙M_{1:T};Z_{ib\_tr}⊙M_{ib\_tr}\right)}}\\ & -\beta \underset{B}{\underset{︸}{I_{\Phi }\left(Z_{ib\_tr}⊙M_{ib\_tr};\left[X_{1:T},X_{T+1:T+F}\right]⊙M_{T+1:T+F}\right)}}, \end{array}$$

*where ⊙ is the element-wise product, also known as the Hadamard dot product, with $M_{1:T}$ and $M_{T+1:T+F}$ representing binary time masks having ones at the indexed time positions, 1:T or T+1:T+F, and zeros at the other time positions. Binary masks $M_{1:T}$ and $M_{T+1:T+F}$ lie in the same manifold, such as the MTS $X_{1:T+F}=Concat\left[X_{1:T},X_{T+F}\right]$, and $M_{ib\_tr}$ in the same manifold, such as the compressed variable $Z_{ib\_tr}$, such that $M_{1:T},M_{T+1:T+F}\in R^{(T+F)\times M}$, $M_{1:T}=Concat\left[1_{T\times M},0_{F\times M}\right]$, $M_{T+1:T+F}=Concat\left[0_{T\times M},1_{F\times M}\right]$; $M_{ib\_tr}$ is a binary mask for the compressed manifold of $Z_{ib\_tr}$ and is filled only by ones: $Z_{ib\_tr}⊙M_{ib\_tr}=Z_{ib\_tr}$.*
*A straightforward approach to designing the compression, described in part A of Equation (A7) for pseudo-images $X_{1:T}$, consists of a convolution Conv and binary convolution BConv, both with strides larger than 2 for spatiotemporal dimension reduction. Without a loss of generality for the explanation of Conv and BConv layers, let us consider the example with one prior time step T=1, $X_{1:T}=X_{1}$, one posterior time step to forecast F=1, $X_{T+1:T+F}=X_{2}$, and 2 spatial dimensions M=2, such that $X_{1:2}\in R^{2\times 2}$; the bottleneck $Z_{ib\_tr}=Z_{1\to 2}$ learns the transition 1→2:*

(A8) $$Z_{1\to 2}=Conv_{\Theta }\left(\left[X_{1},X_{2}\right]⊙M_{1}\right)\in R^{1\times 1\times C}\;\mathrm{and}\;M_{1\to 2}=BConv\left(M_{1}\right)=\left[1\right],$$

*where C is the number of channels for $Conv_{\Theta }$. Proof of Equation (A8) is given in Appendix A. The combination of $Conv_{\Theta }$, BConv with corresponding masks $M_{1}$ and $M_{1:2}$ is usually referred to as* partial convolution $PConv_{\Theta }$ [4], *such that:*

(A9) $$PConv_{\Theta }\left(X_{1:2},M_{1}\right)=\left(Z_{1\to 2},M_{1\to 2}\right).$$

*In a symmetrical approach, still considering the example when T=1,F=1,M=2, the decoding described in part B of Equation (A7) can be designed by a deconvolution DConv and a binary deconvolution BDConv, both with strides of 2, but in this case, the decoder performs as well as a backcast of the prior $X_{1}$ because the deconvolution of $M_{1\to 2}$ returns $M_{1:2}$ instead of $M_{2}$ of B:*

(A10) $$DConv_{\Phi }\left(Z_{1\to 2}⊙M_{1\to 2}\right)=\left[{\tilde{X}}_{1},{\tilde{X}}_{2}\right]\in R^{2}\;\mathrm{and}\;BDConv\left(M_{1\to 2}\right)=M_{1:2}=1_{2\times 2},$$

*and Proof of Equation (A10) is given in Appendix A. The combination of $DConv_{\Phi }$, BDConv with corresponding masks $M_{1\to 2}$ and $M_{1:2}$ is usually referred to as* partial deconvolution $PDConv_{\Phi }$, *such that:*

(A11) $$PDConv_{\Phi }\left(Z_{ib\_tr},M_{ib\_tr}\right)=\left({\tilde{X}}_{1:2},M_{1:2}\right).$$

*When T+F and M are larger than 2, pseudo-images $X_{1:T}$ and masks $M_{1:T}$ must be zero-padded to a square pseudo-image, whose size is a power of 2, and successive PConv and PDConv layers must be used to obtain a 1×1×C bottleneck. For instance, when the padded input image is 254×256, 8 successive PConv and 8 successive PDConv layers must be used, and the model is deep. This forecast model design finally makes use of more traditional CV layers, such as convolutions, compared to classical TS deep models, such as LSTMs; however, the model is directly justified by the IB formulation of the time dimension compression.*

*A drawback of this design is that the binary deconvolution decoder outputs $M_{1:T+F}=1_{(T+F)\times M}$ instead of $M_{T+1:T+F}=Concat\left[0_{T\times M},1_{F\times M}\right]$ from part B of the IB Equation (A7). As a consequence, only with these PConv and PDConv layers would it have to perform a backcast and output an estimation of the prior ${\tilde{X}}_{1:T}$ in addition to the estimated forecast ${\tilde{X}}_{T+1:T+F}$, which means that the bottleneck does not only learn the transition statistics 1:T→T+1:T+F but also the reconstruction of 1:T. This is not the right formulation of the IB bottleneck for MTS forecasting. As a consequence, a skipping connection between the masked input $X_{1:T+F}⊙M_{1:T}$ and the output is then added to the model in order to let all of the information about the prior $X_{1:T}$ pass without any parameter learning, bypassing the bottleneck $Z_{ib\_tr}$. Complemented by these skipping layers, the bottleneck of the network only learns the statistics of the transition between the prior and the forecast. The resulting output for the designed model is then given by:*

(A12)
$$\begin{array}{cc} UNet_{\Phi ,\Theta }\left(X_{1:T+F},M_{1:T}\right) & =\left[X_{1:T}^{IB},X_{T+1:T+F}^{IB}\right]⊙M_{1:T+F}+X_{1:T+F}⊙M_{1:T}\\ \; & =\left[X_{1:T}^{IB}+X_{1:T},X_{T+1:T+F}^{IB}\right], \end{array}$$

*where $UNet_{\Phi ,\Theta }$ is the network with the PConv, PDConv, and skipping layers. $X_{1:T}^{IB}$ is the estimation of the prior $X_{1:T}$ performed through the bottleneck $Z_{ib\_tr}$, whereas $X_{1:T+F}⊙M_{1:T}=X_{1:T}$ is the prior input directly connected to the output by the skipping layer. The output of the network is still not $\left[X_{1:T},X_{T+1:T+F}\right]⊙M_{T+1:T+F}=\left[0,X_{T+1:T+F}\right]=X_{T+1:T+F}$ from part B of the IB loss in Equation (A7), but we will now show the equivalence. With the described design of the prior compression, bottleneck decoding, and prior connection to the output, the upper bound $L_{3}$ loss defined in Equation (7) then becomes equivalent to the following loss on the model estimation output from Equation (A12):*

(A13)
$$L_{3}^{Lap,UNet}\left(\Theta ,\Phi \right)\equiv E_{p_{D}\left(X_{1:T+F}\right)}\left[E_{p_{\Theta }\left(Z_{ib\_tr}|X_{1:T}\right)}\left[{∥X_{1:T+F}-UNet_{\Phi ,\Theta }\left(X_{1:T+F},M_{1:T}\right)∥}_{1}\right]\right].$$

*This equivalence and more details are given in Proof of Equation (A13) of this Appendix A.*

**Proof** **of** **Equation** **(A8).**

(A14) $$\begin{array}{cc} Z_{1\to 2} & =Conv_{\Theta }\left(\left[X_{1},X_{2}\right]⊙M_{1}\right)\\ \; & =Conv_{\Theta }\left(\left[X_{1},X_{2}\right]⊙\left[1_{M},0_{M}\right]\right)\\ \; & =Conv_{\Theta }\left(\left[X_{1},0_{M}\right]\right)\\ \; & =\sigma (\Theta_{1}\times X_{1}+\Theta_{2}\times 0_{M})\in R, \end{array}$$

where σ is a nonlinear activation and Conv is a one-dimensional convolution with parameter $\Theta =[\Theta_{1},\Theta_{2}]$, and:

(A15) $$\begin{array}{cc} M_{1\to 2} & =BConv\left(M_{1}\right)=BConv\left(\left[1,0\right]\right)=\left[1\right], \end{array}$$

where BConv is a one-dimensional binary convolution defined by:

(A16) $$BConv\left(M\right)=Binary\left(Conv_{\left[1,1\right]}\left(M\right)\right)\;\mathrm{and}\;Binary\left(m\right)=\left\{\begin{array}{cc} 1, & \mathrm{if}\;m>0\\ 0, & \mathrm{otherwise} \end{array}\right.$$

□

**Proof** **of** **Equation** **(A10).**

(A17) $$\begin{array}{cc} DConv_{\Phi }\left(Z_{1\to 2}⊙M_{1\to 2}\right) & =DConv_{\Phi }\left(Z_{1\to 2}⊙1\right)=DConv_{\Phi }\left(Z_{1\to 2}\right)\\ \; & =\sigma \left(\left[\Phi_{1}\times Z_{1\to 2},\Phi_{2}\times Z_{1\to 2}\right]\right)=\left[{\tilde{X}}_{1},{\tilde{X}}_{2}\right]\in R^{2}, \end{array}$$

where σ is a nonlinear activation and DConv is a one-dimensional deconvolution of stride 2 with parameter $\Phi =[\Phi_{1},\Phi_{2}]$, and:

(A18) $$\begin{array}{c} BDConv\left(M_{1\to 2}\right)=BDConv\left(\left[1\right]\right)=\left[1,1\right], \end{array}$$

where BConv is a one-dimensional binary convolution of stride 2, defined by:

(A19) $$BDConv\left(M\right)=Binary\left(DConv\left(M\right)\right).$$

□

**Proof** **of** **Equation** **(A13).** $L_{3}^{Lap}\left(\Theta ,\Phi \right)$ is the third component of the upper bound on the IB loss from Equation (6), where $p_{\Phi }\left(X_{T+1:T+F}|Z_{ib\_tr}\right)$ is assumed to be Laplacian, and $L_{3}^{Lap,UNet}\left(\Theta ,\Phi \right)$ is the theoretical loss for our proposed model designed with U-Net and a Laplacian assumption of $p_{\Phi }\left(X_{T+1:T+F}|Z_{ib\_tr}\right)$:

(A20) $$\begin{array}{cc} L_{3}^{Lap}\left(\Theta ,\Phi \right) & =E_{p_{D}\left(X_{1:T+F}\right)}\left[E_{p_{\Theta }\left(Z_{ib\_tr}|X_{1:T}\right)}\left[{∥X_{T+1:T+F}-g_{\Phi }\left(Z_{ib\_tr}\right)∥}_{1}\right]\right]\\ \; & =E_{p_{D}\left(X_{1:T+F}\right)}\left[E_{p_{\Theta }\left(Z_{ib\_tr}|X_{1:T}\right)}\left[{∥X_{T+1:T+F}-X_{T+1:T+F}^{IB}∥}_{1}\right]\right], \end{array}$$

(A21) $$\begin{array}{cc} L_{3}^{Lap,UNet}\left(\Theta ,\Phi \right) & =E_{p_{D}\left(X_{1:T+F}\right)}\left[E_{p_{\Theta }\left(Z_{ib\_tr}|X_{1:T}\right)}\left[{∥X_{1:T+F}-UNet_{\Theta ,\Phi }\left(X_{1:T+F},M_{1:T}\right)∥}_{1}\right]\right]\\ \; & =E_{p_{D}\left(X_{1:T+F}\right)}\left[E_{p_{\Theta }\left(Z_{ib\_tr}|X_{1:T}\right)}\left[{∥X_{1:T+F}-\left[X_{1:T}+X_{1:T}^{IB},X_{T+1:T+F}^{IB}\right]∥}_{1}\right]\right], \end{array}$$

where $X_{1:T}$ and $X_{T+1:T+F}$ are the prior and genuine forecasts given in the training dataset, and $X_{1:T}^{IB}$ and $X_{T+1:T+F}^{IB}$ are the predicted prior and forecast after the compression and decompression using the bottleneck $Z_{ib\_tr}$. Starting with the norm in Equation (A21), we can prove:

(A22) $$\begin{array}{cc} {∥X_{1:T+F}-\left[X_{1:T}+X_{1:T}^{IB},X_{T+1:T+F}^{IB}\right]∥}_{1} & ={∥\left[X_{1:T}^{IB},X_{T+1:T+F}-X_{T+1:T+F}^{IB}\right]∥}_{1}\\ \; & ={∥X_{1:T}^{IB}∥}_{1}+{∥X_{T+1:T+F}-X_{T+1:T+F}^{IB}∥}_{1}, \end{array}$$

and by the linearity of the expected value function:

(A23) $$\begin{array}{c} L_{3}^{Lap,UNet}\left(\Theta ,\Phi \right)+E_{p_{D}\left(X_{1:T+F}\right)}\left[E_{p_{\Theta }\left(Z_{ib\_tr}|X_{1:T}\right)}\left[{∥X_{1:T}^{IB}∥}_{1}\right]\right]=L_{3}^{Lap}\left(\Theta ,\Phi \right), \end{array}$$

such that $L_{3}^{Lap}\left(\Theta ,\Phi \right)\leq L_{3}^{Lap,UNet}\left(\Theta ,\Phi \right)$, because the norm is positive. Moreover, the minimum of $E_{p_{D}\left(X_{1:T+F}\right)}\left[E_{p_{\Theta }\left(Z_{ib\_tr}|X_{1:T}\right)}\left[{∥X_{1:T}^{IB}∥}_{1}\right]\right]$ is 0, obtained when $X_{1:T}^{IB}=0$ for all $X_{1:T}∼p_{D}\left(X_{1:T}\right)$. The two losses, $L_{3}^{Lap,UNet}\left(\Theta ,\Phi \right)$ and $L_{3}^{Lap}\left(\Theta ,\Phi \right)$, share the same minimum. In fact, minimizing $L_{3}^{Lap,UNet}\left(\Theta ,\Phi \right)$ is equivalent to minimizing $L_{3}^{Lap}\left(\Theta ,\Phi \right)$ while forcing the model to not learn a backcast $X_{1:T}^{IB}$; instead, the bottleneck $Z_{ib\_tr}$ only learns the sufficient statistics of transitioning from the prior $X_{1:T}$ to the forecast $X_{T+1:T+F}$. □

## Appendix B. Models

**Table A2:** Model summary of IB-MTS for IRIS data. The encoding part has 7 successive repetitions indexed by $i\in \left[0:7\right]$ of *PConv* and *BatchNormalization* layers followed by *ReLu* activations, except when i=0, no *BatchNormalization* layer is included. The decoding part has 7 successive repetitions indexed by $i\in \left[0:7\right]$ of *UpSampling*, *Concatenation*, *PConv*, and *BatchNormalization* layers followed by *ReLu* activations, except when i=7, no *BatchNormalization* layer is included. EncPConv2D${}_{0}$ is connected to [zero_pad2d${}_{1}$, zero_pad2d${}_{2}$]. DecUpImg${}_{0}$ is connected to [EncReLu${}_{7}$]. DecUpMsk${}_{0}$ is connected to [EncPConv2D${}_{7}$[1]]. DecConcatImg${}_{7}$ is connected to [zero_pad2d${}_{1}$[1], DecUpImg${}_{7}$]. DecConcatMsk${}_{7}$ is connected to [zero_pad2d${}_{2}$[1], DecUpMsk${}_{7}$].

Model: IB-MTS for IRIS Data				
Layer (Type)	Output Shape	Kernel	Param #	Connected to
inputs_img	[(240, 240, 1)]	–	0	[]
(InputLayer)				
inputs_mask	[(240, 240, 1)]	–	0	[]
(InputLayer)				
zero_pad2d${}_{1}$	(256, 256, 1)	–	0	[inputs_img]
(ZeroPad2D)				
zero_pad2d${}_{2}$	(256, 256, 1)	–	0	[inputs_mask]
(ZeroPad2D)				
EncPConv2D${}_{i}$	i=0 (128,128,64) i=1 (64, 64, 128) i=2 (32, 32, 256) i=3 (16, 16, 512) i=4 (8, 8, 512) i=5 (4, 4, 512) i=6 (2, 2, 512) i=7 (1, 1, 512)	7 5 5 3 3 3 3 3	18880 410240 1639168 2361856 4721152 4721152 4721152 4721152	[EncReLu${}_{i-1}$,
(PConv2D)				EncPConv2D${}_{i-1}$[1]]
EncBN${}_{i\neq 0}$				[EncPConv2D${}_{i}$[0]]
(BatchNorm)				
EncReLu${}_{i}$				[EncBN${}_{i}$]
(Activation)				
DecUpImg${}_{i}$	i=0 (2, 2, 512) i=1 (4, 4, 512) i=2 (16, 16, 512) i=3 (32, 32, 512) i=4 (64, 64, 256) i=5 (128, 128, 128) i=6 (256, 256, 64) i=7 (256, 256, 3)	3 3 3 3 3 3 3 3	9439744 9439744 9439744 9439744 3540224 885376 221504 3621	[DecLReLU${}_{i-1}$]
(UpSampling2D)				
DecUpMsk${}_{i}$				[DecPConv2D${}_{i-1}$[1]]
(UpSampling2D)				
DecConcatImg${}_{i}$				[EncReLu${}_{6-i}$,
(Concatenate)				DecUpImg${}_{i}$]
DecConcatMsk${}_{i}$				[EncPConv2D${}_{6-i}$[1],
(Concatenate)				DecUpMsk${}_{i}$]
DecPConv2D${}_{i}$				[DecConcatImg${}_{i}$,
(PConv2D)				DecConcatMsk${}_{i}$]
DecBN${}_{i\neq 7}$				[DecPConv2D${}_{i}$[0]]
(BatchNorm)				
DecLReLU${}_{i}$				[DecBN${}_{i}$]
(LeakyReLU)				
outputs_img	(256, 256, 1)	1	4	[DecLReLU7]
(Conv2D)				
OutCrop	(240, 240, 1)	–	0	[outputs_img]
(Cropping2D)				
Total params: 65,724,969				

**Table A3:** Model summary of LSTM for the IRIS data. The ED-GRU model replaces the LSTM layer with a GRU layer consisting of 240 units and has a total of 347,040 parameters.

Model: LSTM for IRIS Data				
**Layer** **(Type)**	**Output Shape**	**Units**	**Param #**	**Connected to**
inputs_seq (InputLayer)	[(240, 240)]	–	0	[]
slice${}_{0}$ (SlicingOp)	(180, 240)	–	0	[inputs_seq]
lstm (LSTM)	(180, 240)	240	461760	[slice${}_{0}$]
slice${}_{1}$ (SlicingOp)	(60, 240)	–	0	[lstm]
concat (TFOp)	(240, 240)	–	0	[slice${}_{0}$, slice${}_{1}$]
Total params: 461,760				

**Table A4:** Model summary for ED-LSTM for the IRIS data. The ED-GRU model replaces the LSTM layers with GRU layers, each consisting of 100 units. The total number of parameters in the model is 164,100.

Model: ED-LSTM for IRIS Data				
**Layer** **(Type)**	**Output Shape**	**Units**	**Param #**	**Connected to**
inputs_seq (InputLayer)	[(240, 240)]	–	0	[]
slice (SlicingOp)	(180, 240)	–	0	[inputs_seq]
lstm${}_{0}$ (LSTM)	[(180, 100), (100)]	100	136400	[slice]
lstm${}_{1}$ (LSTM)	[(100), (100)]	100	80400	[lstm${}_{0}$[0]]
repeat_vector (RepeatVector)	(60, 100)	–	0	[lstm${}_{1}$[0]]
lstm${}_{2}$ (LSTM)	(60, 100)	100	80400	[repeat_vector, lstm${}_{0}$[0][2]]
lstm${}_{3}$ (LSTM)	(60, 100)	100	80400	[lstm${}_{2}$[0], lstm${}_{1}$[1]]
time_distributed (TimeDistributed)	(60, 240)	240	24240	[lstm${}_{3}$]
concat (TFOp)	(240, 240)	–	0	[slice, time_distributed]
Total params: 401,840				
